# Functional Materials for Fabrication of Carbon-Based Perovskite Solar Cells: Ink Formulation and Its Effect on Solar Cell Performance

**DOI:** 10.3390/ma16113917

**Published:** 2023-05-23

**Authors:** Dena Pourjafari, Nidia G. García-Peña, Wendy Y. Padrón-Hernández, Diecenia Peralta-Domínguez, Alejandra María Castro-Chong, Mahmoud Nabil, Roberto C. Avilés-Betanzos, Gerko Oskam

**Affiliations:** 1 Department of Applied Physics, CINVESTAV-IPN, Antigua Carretera a Progreso Km 6, Merida 97310, Yucatan, Mexico; 2 Facultad de Ingeniería Química, Universidad Autónoma de Yucatán, Periférico Norte, Km 33.5, Chuburná de Hidalgo Inn, Merida 97203, Yucatan, Mexico; 3 Faculty of Science, Universidad Autónoma de San Luis Potosí, Álvaro Obregón 64, Centro 78000, San Luis Potosi, Mexico; 4 Engineering and Science School, Tecnológico de Monterrey, Avenida Eugenio Garza Sada 2501, Tecnológico, Monterrey 64700, Nuevo Leon, Mexico; 5 Facultad de Ingeniería, Universidad Autónoma de Yucatán, Avenida Industrias No Contaminantes por Anillo Periférico Norte, Merida 97203, Yucatan, Mexico; 6 Department of Physical, Chemical and Natural Systems, Universidad Pablo de Olavide, Carretera de Utrera Km 1, 41013 Seville, Spain

**Keywords:** nanoinks, screen printing, inkjet printing, spray deposition, metal oxides, nanomaterials

## Abstract

Perovskite solar cells (PSCs) have rapidly developed into one of the most attractive photovoltaic technologies, exceeding power conversion efficiencies of 25% and as the most promising technology to complement silicon-based solar cells. Among different types of PSCs, carbon-based, hole-conductor-free PSCs (C-PSCs), in particular, are seen as a viable candidate for commercialization due to the high stability, ease of fabrication, and low cost. This review examines strategies to increase charge separation, extraction, and transport properties in C-PSCs to improve the power conversion efficiency. These strategies include the use of new or modified electron transport materials, hole transport layers, and carbon electrodes. Additionally, the working principles of various printing techniques for the fabrication of C-PSCs are presented, as well as the most remarkable results obtained from each technique for small-scale devices. Finally, the manufacture of perovskite solar modules using scalable deposition techniques is discussed.

## 1. Introduction

Since the first report in 2009 [1], the popularity of photovoltaic (PV) devices known as perovskite solar cells (PSCs) has skyrocketed due to their many advantages, such as the utilization of a low-cost, and earth-abundant hybrid lead halide perovskite with ambipolar transport properties [2], low-exciton binding energy, long free-charge diffusion length, bandgap tunability, and highly efficient light absorption [3,4].Remarkably, in less than 15 years, their power conversion efficiency (PCE) has increased from 3.81% [1] to 25.7% [5] in single junction cells, making them a promising complement to the currently dominating crystalline silicon-based solar cells [6]. The major drawbacks of conventional PSCs are related to low chemical stability, expensive materials, and toxicity. Degradation of the devices is caused by the chemical instability of the organic hole transport layer (HTL), metallic top electrode, and the hybrid perovskite layer upon exposure to UV or high temperature, and incorporation of oxygen and moisture during working cycles. For instance, the metal contact (gold, silver, or aluminum) may migrate into the perovskite material through the HTL after thermal stress [7]. To overcome such drawbacks and simultaneously improve the device efficiency, researchers have explored various methods, including the incorporation of electron/hole conducting nanoparticles (NPs) into the electron transport layer (ETL) and hole transport layer, modification of fabrication processes, engineering of the ETL/perovskite and perovskite/HTL interfaces, altering the perovskite precursor formulation, using hydrophobic HTL and top contact materials, and more effective sealing and encapsulation.

Among various architectures of perovskite solar cells (PSCs), the carbon-based perovskite solar cell (C-PSC) is an interesting architecture due to the low-cost, easy, scalable, and fully printable manufacturing process. The hydrophobicity of carbon materials as a top contact can significantly improve the stability of the photovoltaic device. This type of solar cell consists of a patterned transparent conductive oxide substrate (TCO), a blocking, compact layer (BL or CL), a mesoporous metal oxide as an electron transport layer (m-ETL), either a large bandgap metal oxide as an insulating separator layer or a hybrid perovskite layer, and a (mesoporous) carbon layer as a top contact; the mesoporous layers are impregnated with the hybrid perovskite absorber material. Depending on the cell architecture, one or two layers may or may not be present in the structure.

**Figure 1:**
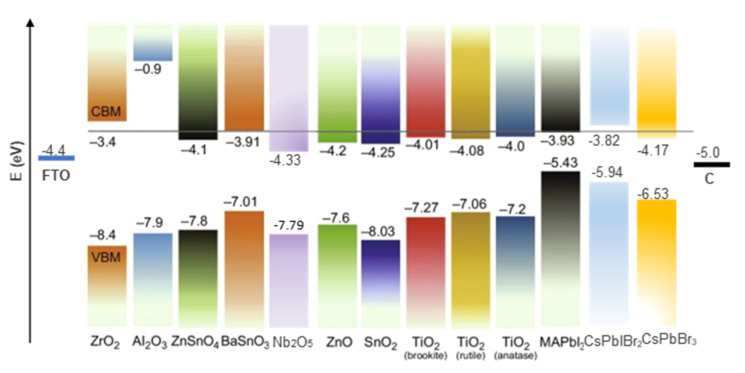
Energy band diagram of various metal oxides used in perovskite solar cells with carbon electrode. Reproduced with permission from Ref. [8].

For instance, the m-ETL and/or the insulating layer are absent in the planar architecture, in which the BL acts as an ETL. Additionally, extra layers can be incorporated between the m-ETL and the perovskite layer, or between the perovskite and carbon layers to improve the device performance. C-PSCs are divided into two types: low-temperature and high-temperature. In low-temperature C-PSCs, a carbon paste with low curing temperature (<120 °C) is deposited after perovskite deposition and crystallization. In contrast, in high-temperature C-PSCs, a carbon paste is deposited and sintered at 400 °C. Then, after cooling down to room temperature, the perovskite precursor solution is deposited by drop casting and further annealed. Independent of the process fabrication temperature, the working principle of the C-PSCs is as follows: photogenerated electrons in the conduction band (CB) of the perovskite material are injected into the CB of the ETL metal oxide, while holes are extracted from the perovskite valence band (VB) by the carbon electrode. For efficient charge injection, transport, and extraction, the energy levels of the different functional layers in the cell structure must be compatible. Figure 1 shows the energy band positions of the fluorine-doped tin oxide (FTO) substrate, different electron extraction materials (ETMs), insulator layers, perovskite materials, and carbon top contact, which are mentioned in this review. 

**Figure 2:**
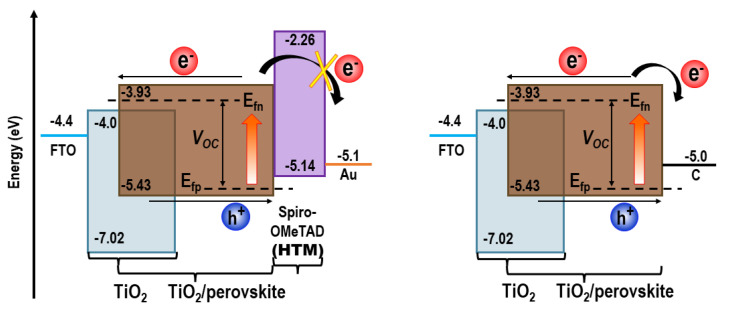
Conventional PSC (**left**) and C-PSC (**right**) working principles upon light illumination. E_fn_ and E_fp_ represent the electron and hole quasi-Fermi levels, respectively. Reproduced with permission from Ref. [11].

The C-PSC has achieved more than one-year stability [9,10]; however, the cell performance is not as good as other PSC architectures. Chen et al. [11], have explained several principal issues related to the lower performance of C-PSCs compared to conventional PSCs with HTL and metal contact. Briefly, there are three main reasons for lower performance of C-PCS, which are lower open circuit potential (V_OC_), slower hole transfer, and serious electron back transfer to the carbon electrode. The V_OC_ is related to the difference between the electron and hole quasi-Fermi levels in the perovskite film. Various research has shown that the recombination losses occur mainly at the perovskite/ETM or perovskite/hole transport material (HTM) interfaces or close to the contacts rather than in the bulk. This can be interpreted that the electron and hole quasi-Fermi levels (E_fn_ and E_fp_) in the perovskite film can be affected by band bending at the perovskite/ETM or HTM interfaces resulting in voltage losses. For the C-PSCs, the band bending can be affected by carbon electrode [12,13]. The Fermi level of carbon is usually higher than the highest occupied molecular orbital (HOMO) level of HTMs, thus the E_fp_ in C-PSCs stands at higher energy resulting in a lower V_OC_. Slower hole transfer in C-PSCs is due to the lower hole selectivity at the perovskite/carbon interface. As shown in Figure 2**,** the difference between the Fermi-level of carbon and valence band level of the perovskite is larger than the difference between the HOMO level of the HTM and the VB level of the perovskite, which slows down the hole transfer step. In HTM-based PSCs, the lowest unoccupied molecular orbital (LUMO) level of the HTM is found at higher energy than the conduction band (CB) level of the perovskite, which could suppress the electron back transfer from the perovskite to the metal electrode. However, in C-PSCs, due to the positions of the energy levels, the charge separation rate at the perovskite/carbon interface is slower which may result in higher charge recombination and charge accumulation and, hence, larger hysteresis in the current density-voltage (J-V) curves. 

Therefore, one of the main objectives of research on C-PSCs is to achieve higher V_OC_, and to enhance charge separation, extraction, and transport properties. These goals can be achieved by using novel ETMs, modifying the existing materials or using novel inorganic HTMs and/or cost-effective HTLs between the perovskite and carbon electrode. 

One important ETM in solar cell structure is mesoporous inorganic metal oxide. The most studied mesoporous metal oxide layer is titania (TiO_2_) due to its extensive development in the field of dye-sensitized solar cells. TiO_2_ has a wide band gap, and it can be synthesized in different morphologies such as nanoparticles, nanorods, nanotubes, nanosheets, etc. Titania is commercially available in the market in the form of nanopowder, paste, and colloid and many research groups purchase it for the fabrication of electronic devices. Three big companies supply the commercial pastes typically used in third generation solar cells: Greatcell Solar Materials (formerly part of Dyesol) [14], Solaronix [15], and WonderSolar [16]. However, to improve the electronic properties of titania ETLs and enhance the TiO_2_/perovskite interface for a better match between the energy levels, researchers have synthesized titania nanopowders and have prepared homemade pastes. This allows to study the effect of different titania crystallographic phases, nanoparticle sizes and doping with metallic nanoparticles on the solar cell performance.

Another important layer in C-PSCs is the carbon electrode, which can be deposited using commercially available pastes. A common carbon paste consists of carbon black (CB), graphite, and organic binder material. Graphite powder provides the electrical conductivity of the carbon layer, and the morphology can be spherical or flake-shaped within the size range of 10–20 μm [17]. The carbon black nanoparticles typically have diameters around 30 nm and improve the interconnection between graphite sheets and, therefore, the hole extraction efficiency and conductivity [18]. The organic binders in the carbon paste provide adequate viscosity and adhesion of the carbon to the substrate. Commonly, ethyl cellulose, hydroxypropyl cellulose, and terpineol, are used as binder and adhesive, respectively. To enhance adhesion to the underneath separator layer (zirconia or alumina), small amounts of zirconia or alumina powder are usually considered in the paste formulation. To further improve the electrical properties at the perovskite/carbon interface, organic binders, and carbon NPs with different morphologies (such as single-walled and multi-walled carbon nanotubes [SWCNTs and MWCNTs], graphene, and carbon from biomass) have been incorporated [19,20,21,22,23,24,25]. Also, to enhance carbon conductivity, several researchers have added inorganic nanoparticles (NP) into their homemade carbon pastes [26,27,28,29,30]. 

To fabricate C-PSCs several printing techniques such as screen printing, inkjet printing, spin coating, slot-die coating, blade coating and spray coating have been used. Each technique has its own advantages and disadvantages. For example, the most efficient PSCs have been manufactured using spin coating; however, this technique is mainly suitable for small scale and batch fabrication. In contrast, screen printing, spray, blade coating and slot-die coating have been used in the manufacture of large photovoltaic devices, although with lower performance due to the imperfection and defects of the final deposited layers. The reports on fully printed devices by only one technique are few. Usually, for the fabrication of the entire C-PSCs more than one printing technique is used. This is due to the fabrication and operational restrictions of some printing techniques. For example, preparation of aqueous binder-free pastes suitable for screen-printing is still challenging and this technique is particularly used for deposition of non-aqueous viscous pastes. Another example is the inkjet printing technique in which the size of dispersed nanoparticles of the ink is critical to avoid nozzle blockage. Carbon pastes with micron-sized graphite sheets are not suitable to be used in this technique. In spin-coated devices, commonly all layers are deposited using this technique except the carbon layer which is deposited by screen printing, blade or brush painting and spray coating. In screen-printed devices the blocking layer is mostly deposited by spray pyrolysis and the perovskite layer by drop-casting. The choice of the printing technique highly depends on the rheological properties of the original ink, paste or colloid and the solvent in which the nanoparticles are dissolved or dispersed. 

Fluid characterization may help with the right selection of printing technique. Different characterization techniques and equipment exist, such as rheology to determine fluid rheological properties (yield stress, relaxation time, thixotropic properties, etc.), viscosimetry to quantify fluid viscosity, and dynamic light scattering (DLS) to determine the size and size distribution of molecules and particles in a dispersion before layer printing. As mentioned before, these photovoltaic devices are composed of a stack of different hydrophilic (inorganic metal oxide) and hydrophobic (carbon electrode) layers. The light absorbing material, i.e., the hybrid perovskite layer must be deposited on and/or infiltrated into the layers. An appropriate interfacial contact between the perovskite and other layers is required for good performance of the device. To measure the wettability of the layers for the perovskite precursor solution, contact angle measurement is a useful technique that can be used after printing of the layers. The next step, after selection of a suitable ink, is to optimize the operational parameters of each printing technique in order to achieve uniform and pinhole-free layers. 

In this review, we report on printing techniques for the deposition of functional materials for the manufacture of carbon-based perovskite solar cells. We provide important information on the principles of different printing techniques and their operational parameters. In the following sections, we focus on the extensive research literature reporting recipes and formulations for the preparation of pastes, inks, and colloids of different layers in C-PCSs. In this review, we consider neither the formulation of the perovskite precursor solution, which is covered in many reviews, nor the perovskite deposition method, which is generally drop casting or spin coating. We exclude those reports that use commercial pastes since it is beyond the scope of this review. We highlight research that achieved the fabrication of efficient C-PSCs by modification of homemade printing inks, pastes and colloids. Finally, we will mention the reports on fabrication of this type of solar modules and panels, emphasizing the scale-up opportunities and commercialization feasibility of these potentially low-cost devices. 

## 2. Printing Techniques

### 2.1. Spin Coating 

The spin coating technique produces thin, consistent coatings, which has enabled the quick manufacture of effective and reliable PSCs. An extensive variety of coating solutions and substrates can be used in this technique. The thickness of the coating can be controlled from a few nanometers to a few micrometers. The final coating properties such as thickness and homogeneity depend on the fluid rheological properties such as viscosity and thixotropy as well as surface tension. Other experimental parameters such as dispense volume, solution concentration, spin speed and time, and acceleration/deceleration rate impact the final coating properties [31,32].

**Figure 3:**
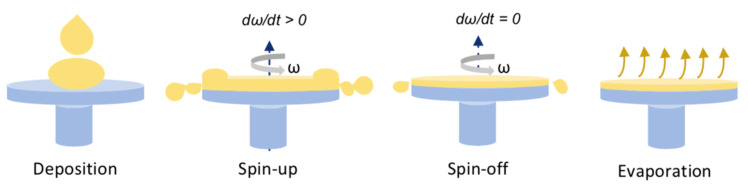
Schematic view of the spin coating stages (ω is the angular velocity).

The spin coating process consists of four main steps: deposition, spin-up, spin-off, and evaporation, which are shown in Figure 3 [33]. During the deposition step, a solution falls on a rotating substrate (dynamic spin coating) or fixed substrate (static spin coating) typically using a pipette or syringe. The solution spreads on the substrate due to the centrifugal or gravitational forces and, depending on its rheological properties, it may cover or wet the substrate completely. During the spin-up, the substrate is accelerated up to its desired rotational speed. At the very beginning, the substrate rotates faster than the fluid. While rotating, the fluid is radially driven out from the substrate due to the centrifugal forces. When the substrate reaches its desired speed, the fluid rotates at the same speed as the substrate and the film thickness decreases. In the third stage, the fluid rotates at a constant rate, flows out to the substrate perimeter, and continues thinning gradually. If the fluid contains a volatile solvent, usually it is possible to observe a color change in the deposited film. Finally, the fluid spin-off is stopped, and the thinning is dominated by solvent evaporation and film drying [32,33].

To our knowledge, the highest reported efficiency for HTM-free C-PSCs fabricated by the spin coating technique (except the carbon layer) is 17.42%, and corresponds to the following architecture: BL-TiO_2_/m-TiO_2_/MAPI/C(commercial paste) [34]. The highest efficiency for a cell with insulating layer is 9% and corresponds to BL-TiO_2_/m-TiO_2_/ZrO_2_ (homemade paste)/MAPI/C (homemade paste) structure [35].

As explained previously, fluid rheology has a strong influence on the quality of the deposited layer. For photovoltaic devices fabricated by spin coating, a choice of solvent for ink preparation is crucial and depends on the nature of the solute. For example, to deposit the blocking layer, a molecular ink is generally used in which the precursor is completely dissolved. For metal oxide mesoporous ETL or the insulator layer, a nano-ink is formulated in which the nanoparticles are dispersed into the appropriate solvent [36,37]. In the most common spin-coated C-PSCs, the insulating layer is not present, and the structure is FTO/BL or m-ETL/perovskite/carbon. However, as mentioned before, the carbon layer is usually not deposited using this technique. For this reason, in the following sections we present examples of reports in which the blocking and electron transport layers have been modified to achieve better performance. Finally, the work that reported strategies to improve the interface between perovskite and carbon are worth mentioning. 

#### 2.1.1. Blocking Layer

The research to improve the blocking layer goes back to the fabrication of dye-sensitized solar cells and is a well-known process. The blocking layer must be compact and pinhole-free. It is mostly TiO_2_, but sometimes SnO_2_ or ZnO are also used as a blocking layer [38,39]. A TiO_2_ compact layer (c-TiO_2_) usually is deposited from a normal TiO_2_ sol or a molecular solution containing a titanium precursor such as titanium isopropoxide (TTIP), titanium diisopropoxide bis acetylacetonate, titanium diisopropoxyacetate, titanium(diisopropoxide) bis(2,4-pentanedionate), and bis(2,4-pentanedionato)-bis(2-propanolato) titanium (IV) in ethanol, isopropyl alcohol (IPA), and 1-butanol [40,41,42,43,44,45,46,47,48,49,50]. To improve the electron transport properties of the blocking layer, doping with several metallic elements has been suggested [47,51,52,53]. Zhu et al. first deposited a compact titania layer of approximately 60 nm from a titania sol. After sintering, a solution of CsBr in deionized water (DI): IPA was directly spin-coated on the compact layer. The CsBr acts as a modifier resulting in an increase in the conduction band minimum (CBM) of the TiO_2_ ETL from −4.00 to −3.81 eV and, hence, a decrease in the work function, thus promoting favorable band alignment at the ETL/perovskite heterojunction. This doped compact layer suppresses recombination and improves charge carrier extraction and transport due to the band alignment [47]. In other work, Zhang et al. suggested Co/Eu doping of the titania compact layer. First, the TiO_2_ sol was obtained using a TTIP precursor solution. After the formation of titania nanoparticles, different molar ratios of Eu(NO_3_)_3_.6H_2_O/TiO_2_ and Co(NH_3_)_6_]Cl_3_/TiO_2_ were mixed, and spin coated on the FTO substrate to obtain a Co/Eu-TiO_2_ compact layer. They demonstrated that Co doping decreases the trap density in the TiO_2_ ETL and up-shifted the band edges of TiO_2_, while Eu doping increased the photo-response in the UV region. Combining them, the performance of the device with Co/Eu co-doped TiO_2_ was appreciably enhanced compared to the solar cells based on an undoped TiO_2_ BL, leading to a 26.4% increase in efficiency [51].

#### 2.1.2. Electron Transport Layer

A suitable TiO_2_ ink for spin coating is commonly obtained from a commercial paste diluted in ethanol [40,41,54,55,56,57,58]. The paste consists of the spherical nanoparticles of titania anatase phase synthesized by the sol-gel method. However, many researchers have investigated the effect of different morphologies and particle sizes on solar cell performance by preparing homemade pastes. Acchutharaman et al. synthesized different sizes and porosities of TiO_2_ nanospheres via a solvothermal route by varying the concentration of acetic acid. Adequate porosity of the nanoparticles forming the ETL facilitates better crystallinity of the perovskite layer. The nanoparticle size (NPs) plays an important role in the back-scattering properties of the ETL. Larger particles scatter a larger number of photons, resulting in more photons that may be absorbed by the perovskite material and, therefore, the generation of a high photocurrent in the device. Also, lower degrees of crystallinity of the NPs was shown to lead to a larger number of electron-trapped oxygen vacancies. In this study, the titania layer with largest nanoparticles with an average diameter of approximately 17 nm and better light-harvesting properties, acts as a suitable scaffold to perovskite crystal growth resulting in a decrease in recombination and an increase in short-circuit current compared to nanoparticles with an average diameter of 12.5 nm. From impedance spectroscopy (IS), the highest value for the recombination resistance (R_rec_) was obtained for the solar cells with the largest particles suggesting that these cells transfer the charge carrier effectively with very low non-radiative recombination at the interfaces of the device. Therefore, cells based on the ETL with 17 nm particle size show a lower recombination rate and higher V_OC_ with a champion efficiency of 10.6% compared to the ETL with 12.5 nm particle size with a champion efficiency of 8.2% [59].

Another modification of the titania ETL in carbon-based perovskite solar cells is changing the crystalline phase. There are four commonly known polymorphs of titania found in nature, i.e., anatase (tetragonal), rutile (tetragonal), brookite (orthorhombic), and TiO_2_-B (monoclinic) [60]. Rutile is the most stable polymorph of TiO_2_ in the bulk at all temperatures, exhibiting lower total free energy than metastable phases of anatase and brookite. Anatase is the most used polymorph for solar cell applications because of its potentially higher conduction band edge energy and lower recombination rate of electron−hole pairs [61]. All these phases have been used in the structure of the PSCs as an ETL. Figure 1 shows the energy level of different phases in alignment with MAPbI_3_ [8].

There are various reports on the incorporation of the other titania phases into the solar cell architecture. Xiao et al. reported on the rutile passivation of an anatase TiO_2_ scaffold by introducing forming an anatase/rutile mixed phase system for carbon-based perovskite solar cells. The rutile phase was formed during the hydrolysis process by immersion of the anatase films, which were spin coated on the FTO substrate, into the TiCl_4_ aqueous precursor solution with different concentrations. The mixed phase exhibited higher conductivity, faster charge extraction, and reduced charge recombination by passivating defects at the interface of TiO_2_/perovskite films, which resulted in a higher open circuit voltage up to almost 1000 mV and champion efficiency of 15.21%. In addition, benefiting from the stable and better ultraviolet light filtration properties of the rutile phase, devices exhibited excellent stability maintaining 92% of their initial efficiency after 200 days of storage in ambient air [62].

**Figure 4:**
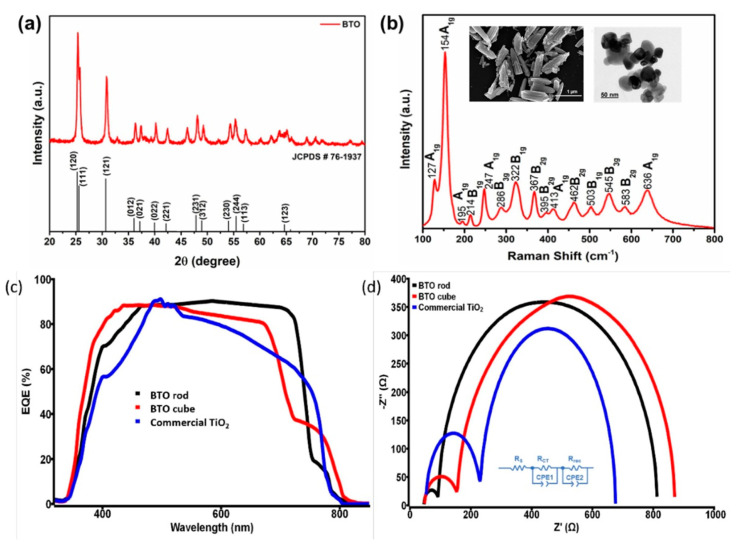
(**a**) X-ray diffraction pattern; (**b**) Raman spectrum with inset of SEM image of brookite nanorods and TEM image of brookite nanocubes; (**c**) EQE of the champion devices comparing brookite nanorods and nanocubes with commercial anatase; and (**d**) IS representation along with the fitted electrical circuit diagrams. Reproduced with permission from Ref. [63].

Also, a brookite phase has been used in the structure of carbon-based perovskite solar cells and was reported in the work of Bhandari et al. [63]. Brookite nanorods and cubic nanoparticles were synthesized (inset of Figure 4), and the spin coating technique was used to deposit the brookite colloidal suspensions in water onto the substrate. X-ray diffraction spectra (XRD) was used to determine the phase purity of the synthesized material (Figure 4a). The peaks at 25.34°, 31.5°, 36.2°, 40°, 47.5° and 55.5° correspond to the (120), (111), (121), (012), (022), (231) and (244) planes respectively and are the major characteristics planes for brookite TiO_2_ (BTO). Raman spectra was conducted to confirm XRD analysis and dismiss the possibility of overlapping of anatase at 25.28° and reflection of brookite at 25.34°. Figure 4b shows that all Raman peaks match the literature in the range of 100 cm^− 1^ to 700 cm^− 1^ which clarifies the absence of anatase, rutile and any other titanium oxide or impurities. The film thickness was adjusted through the number of coating steps. The brookite-based cells reached a champion PCE of 15% and a higher open-circuit voltage of more than 1000 mV compared to the commercial anatase titania-based cells. The solar cells composed of the nanorod shaped brookite particles show better photovoltaic performance compared to those consisting of cubic particles and commercial titania. This may be due to the large surface area of nanorods, as illustrated by nitrogen physisorption measurements. The larger surface area enhances the cohesion of the ETL/perovskite contact, lowering the number of surface defect traps and refining the morphology of perovskite. This also was confirmed by incident photon-to-current conversion efficiency (IPCE) measurements showing better coverage in the region of ~500 to 780 nm and hence a higher electron transfer rate for brookite nanorod devices due to the better quality of the perovskite/brookite interfaces (see Figure 4c).

In order to obtain more details on the charge transfer (R_Ct_), charge recombination (R_rec_), and inner resistances they carried out impedance spectroscopy. According to the IS spectra (Figure 4d), a smaller loop in the high-frequency region was obtained for the nanorod shape brookite which suggests a lower R_Ct_ value indicating a high charge transfer rate between the transport layer and perovskite. This means higher values of FF and J_sc_ for nanorod brookite-based devices. On the other hand, a large loop with a high value of R_rec_ explains a lower rate of charge recombination, increasing the V_OC_ of the device. Hence, they concluded that the type and morphology of the active layer greatly altered the electrical transport properties of the device demonstrating the importance of grain boundary trap passivation via structural modulation of nanomaterials, which can hugely improve the performances of devices [63].

As mentioned previously, replacing the commonly used titania ETL in C-PSCs with another metal oxide is one way to improve the cell performance and overcome several disadvantages of titania such as its low bulk electron mobility (<1 cm^2^ V^−1^ s^−1^), structural defects acting as charge traps, the high UV-photocatalytic activity resulting in decomposition of organic compounds, and high processing temperature [60,64].

Usually, a very thin layer of SnO_2_ is spun onto the conductive glass substrate from a SnO_2_ colloidal dispersion in deionized water [65,66,67,68,69,70], followed by spin coating of the perovskite layer. In many reports, the inorganic CsPbIBr_2_ perovskite is used in SnO_2_-based devices because of the better compatibility of its conduction band edge energy with inorganic perovskite (see Figure 1) [66,71]. There are different strategies to improve the performance of SnO_2_-based photovoltaic devices by modifying the SnO_2_ layer in order to engineer the charge transport properties at the tin oxide/perovskite interface. 

**Figure 5:**
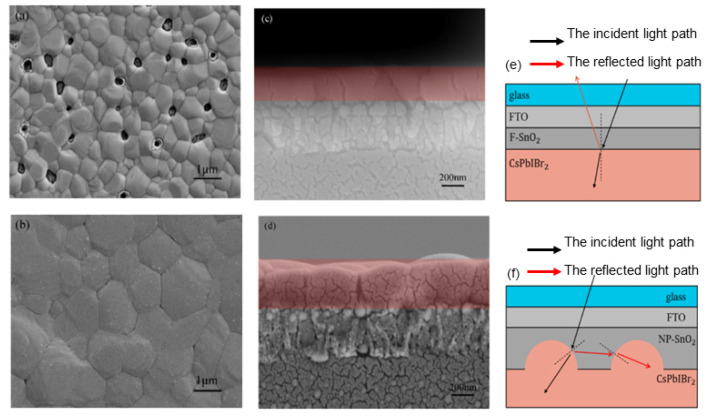
SEM images of (**a**) CsPbIBr_2_ cell based on F–SnO_2_ film; (**b**) CsPbIBr_2_ cell based on NP-SnO_2_ film; (**c**) cross section of CsPbIBr_2_ film based on F–SnO_2_ film; (**d**) cross section of CsPbIBr_2_ cell based on NP-SnO_2_ film; (**e**) CsPbIBr_2_ based on F–SnO_2_ film; (**f**) the light path in the CsPbIBr_2_ cell based on NP-SnO_2_ film. Reproduced with permission from Ref. [67].

For example, this can be achieved by improving the SnO_2_ porosity or reducing the surface defect density. He et al. showed that the introduction of nanopores into the SnO_2_ film can improve the performance of the carbon-based PSCs. They obtained the nanoporous SnO_2_ film by spin coating the tin oxide colloidal solution aided by monodisperse polystyrene microspheres (PS-MS). The nanoporous structure provides more electron-transporting pathways, increases the electronic transmission efficiency, and reduces the charge transport resistance (R_ct_) of the PSCs. In addition, the nanoporous structure improves the crystallinity of the perovskite upper layer and reduces defects in the film. Also, the nanoporous structure can change the refraction of the incident light, and the refracted light can be introduced into the perovskite film (Figure 5e,f). Therefore, nanoporous SnO_2_ improves the absorption capacity of the PSCs by reducing the reflection of the light [67].

To reduce surface defects, Qiang et al. obtained a tin oxide and lithium-doped tin oxide precursor solution via a simple low-temperature sol-gel method and subsequently deposited onto the substrate using spin coating. The authors compared the SnO_2_ with Li-SnO_2_ ETL in carbon-based perovskite solar cells. They explained that Li^+^ doping causes SnO_2_ lattice defects and forms a mixed valence state structure in which Sn^2+^/Sn^4+^ coexist, which can effectively improve the conductivity of SnO_2_. This approach offers two advantages over using a pure SnO_2_ ETL: it provides additional valence electrons to the SnO_2_ lattice and increases the electron extraction ability of ETL resulting in a 42.3% improvement in PCE compared to the non-doped SnO_2_ ETL [72]. In addition to TiO_2_ and SnO_2_, other inorganic electron transport layers have been explored in PSCs structure such as zinc oxide (ZnO), niobium pentoxide (Nb_2_O_5_), Zn_2_SnO_4_ (ZSO), BaSnO_3_ (BSO), amorphous InGaZnO_4_ and etc. [60,64,73,74,75,76].

**Figure 6:**
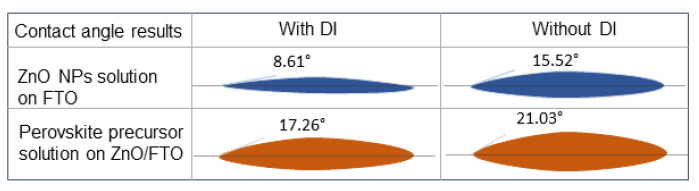
Schematic view of the contact angles of ZnO NPs solution dropped on FTO substrates with DI treatment and without DI treatment and the contact angles of the perovskite precursor solution dropped on ZnO/FTO films with and without DI treatment.

ZnO is comparable to titania, with a similar energy band alignment with perovskite, but with a much larger electron mobility. Fu et al. proposed a feasible interface engineering method using deionized (DI) water to treat the FTO substrate (Figure 6) [77]. This enhances the wettability of the FTO substrate and improves the ZnO nanoparticles spreading on FTO. ZnO nanoparticles were obtained using low-temperature synthesis and were spun onto the DI water-coated-FTO substrate. Using contact angle measurements, they illustrated that the quality of the perovskite layer directly depends on the quality of the ZnO ETL, which in turn is significantly related to the quality of the FTO substrate. Using the pre-treatment of the substrate, they obtained a smooth and uniform perovskite layer with fewer pinholes and larger grains, with a champion PCE of 12.39% compared to the non-treated FTO substrate with PCE of 10.95%. 

**Figure 7:**
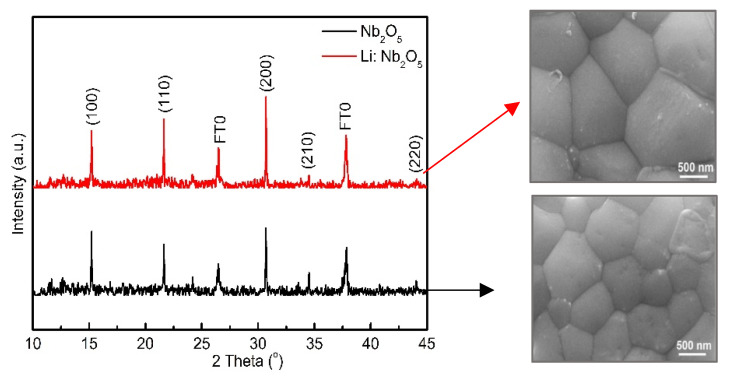
XRD spectra of the CsPbBr_3_ films prepared on different ETLs with corresponding SEM image of CsPbBr_3_ film grown on Nb_2_O_5_/FTO and Li: Nb_2_O_5_/FTO. Reproduced with permission from Ref. [74].

The last but not the least ETL modification in carbon-based perovskite solar cells is the incorporation of Nb_2_O_5_ and Li-doped Nb_2_O_5_. Zhao et al. reported a high open circuit voltage of 1.505 V using Li-doped Nb_2_O_5_ as an electron transport layer in C-PSCs [74]. They used spin coating method to dope a sputtered layer of Nb_2_O_5_ on FTO with lithium using acetonitrile solutions of 0.025 M, 0.05 M, and 0.075 M lithium bis(trifluoromethanesulfonyl)imide (Li-TFSI). As in previous work, they also mentioned the importance of the nature of the substrate on the quality of the perovskite layer. The inorganic CsPbBr_3_ average grain size on Li: Nb_2_O_5_/FTO was larger than on Nb_2_O_5_/FTO, which was 0.98 mm and 0.78 mm respectively (Figure 7). A better and uniform distribution of perovskite precursor solution on Li: Nb_2_O_5_/FTO resulted in an improvement in perovskite crystallinity, promoting light capture and charge transport and therefore, better performance of the device. From Hall effect measurements, the electron mobility and conductivity for Nb_2_O_5_ and Li: Nb_2_O_5_-based devices were found to be 15.7 and 33.8 cm^2^ V^−1^ s ^−1^ and 1.5 × 10^−5^ and 2.6 × 10^−5^ S cm^−1^ respectively. This indicates that Li-doping increases the electronic conductivity of the amorphous Nb_2_O_5_, which facilitates better charge extraction and lower recombination in the photovoltaic devices. 

#### 2.1.3. Perovskite/Carbon Interface

The present section shows the strategies that have been proposed to improve the interface between the perovskite layer and carbon electrode by incorporating carbon nanoparticles or nanotubes as well as inorganic nanoparticles during perovskite deposition. In C-PSCs, the PCE depends not only on the energy level alignment between perovskite and carbon but also on the quality of the interfacial connection between these layers to guarantee a perovskite/carbon interface without pinholes. This can be achieved by adding carbon nanomaterials into the perovskite precursor solution in order to obtain a uniform, pinhole free and less rough perovskite layer with good optical quality and high crystallinity. In the following paragraphs we will report on several proposed strategies in which a spin coating ink of perovskite precursor mixed with carbon materials was formulated to achieve (1) an improvement in perovskite crystal growth, and (2) a better connection between perovskite crystals and the top carbon electrode deposited by blade coating. 

**Figure 8:**
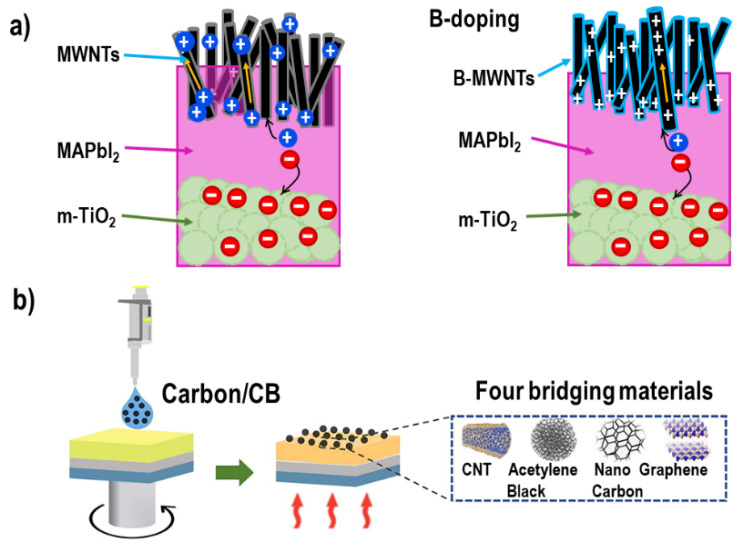
Schematic of perovskite cell fabrication processes using different nanomaterials dispersed in the perovskite layer. (**a**) B-MWNTs on PbI_2_ layer followed by MAPbI_3_ perovskite film, reprinted with permission from [78]; Copyright {2017} American Chemical Society. (**b**) CNPs dispersed in the antisolvent as chlorobenzene; reproduced with permission from Ref. [79].

In 2017, Zheng et al. deposited boron doped multi-walled carbon nanotubes (B-MWNTs) onto the PbI_2_ layer followed by annealing to obtain MAPbI_3_ perovskite film (See Figure 8a) [78]. The incorporated boron increases the work function, carrier concentration, and conductivity of MWNTs. Therefore, C-PSCs prepared with B-MWNTs showed an enhancement in the hole extraction efficiency and transport dynamics of the counter carbon electrode resulting a PCE improvement from 10.70% (cell with the undoped MWNTs) to 14.60% due to lower number of defects and higher conductivity. In 2019, Junshuai Zhou et al. added small amounts of multi-walled carbon nanotubes (MWCNT) (0.5% and 1.0% in volume fraction) to the FAI:MABr precursor solution [21]. The incorporation of MWCNTs as templates in the perovskite precursor helps to fill the boundary between perovskite grains and reduce the transformation kinetics of the perovskite, which benefits defect-free crystal growth. The results showed an improvement of the perovskite morphology, grain size and crystallinity with a long-term stability of 93% under ambient air conditions for 22 weeks.

**Figure 9:**
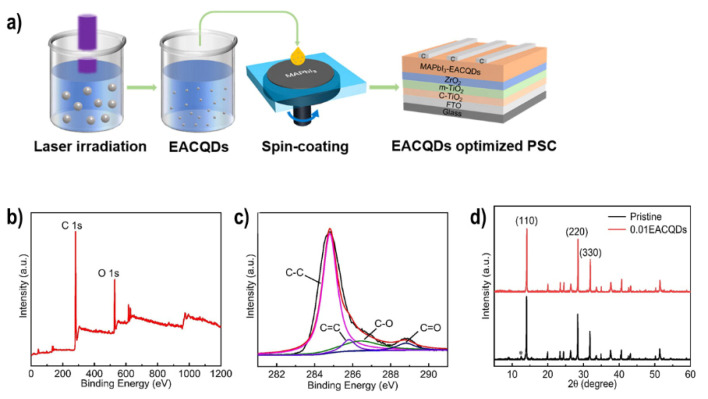
(**a**) CQDs dispersed in the antisolvent as ethyl acetate, (**b**) XPS spectra of EACQDs, (**c**) deconvolution of C, and (**d**) XRD image of pristine and 0.01 EACQDs optimized PSCs, reproduced with permission from Ref. [80].

Gao et al. used chlorobenzene as antisolvent with four different types of carbon materials (See Figure 8b), including CNTs (particle size, 5–15 nm), acetylene black (AB, particle size, 30–40 nm), nanocarbon (NC, particle size, 40–50 nm), and graphene (particle size, 3–5 µm) [79]. They found that the order of crystallinity was graphene > CNT > AB > NC, which meant that graphene had the best graphitization and that the NC was amorphous. As a result, the conductivity of graphene was better than for the other materials. However, the results indicated that the CNTs had a better interfacial contact effect and work function, leading to a PCE of 15.09%. They argue that the morphology of the interfacial bridging of the carbon materials played a more important role than the energy band alignment and conductivity. Also, carbon quantum dots (CQDs) have been used as interconnecting agents between perovskite and carbon electrode. In 2021, Li et al. fabricated C-PSCs using ethyl acetate including CQDs as anti-solvent (See Figure 9a) [80]. The CQDs were obtained by laser irradiation and the color change of the colloidal solution from dark to light yellow indicated the formation of the product. X-ray photoelectron spectroscopy (XPS) was used to reveal the surface chemistry of EACQDs as shown in Figure 9b. C 1s and O 1s were found in the whole spectrum of EACQDs. The deconvolution of C was conducted and displayed in Figure 9c. The peaks located at about 284.8, 285.8, 286.3, and 288.8 eV can be assigned to C–C, C=C, C–O, and C=O, separately. The EACQDs inside the spin-coated perovskite film passivate the defects, while perovskite crystallization results in a slower carrier recombination rate and faster carrier transportation, XRD results of pristine and EACQDs optimized PSCs are shown in Figure 9d.

The role of CQDs also was confirmed by Xu et al., who showed that the presence of this material in deposited perovskite layers results in the formation of larger size perovskite grains and excellent crystallinity [81].

### 2.2. Screen Printing 

**Figure 10:**
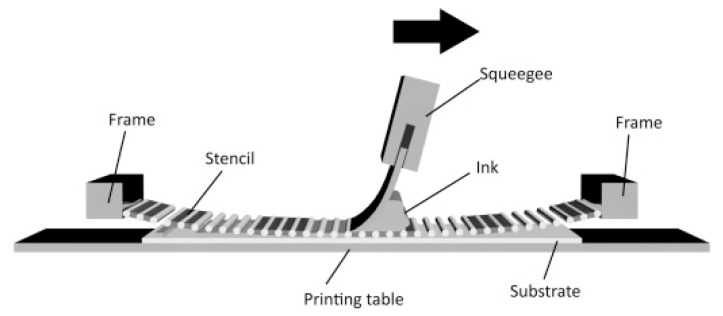
Schematic illustration of the basic principles of screen printing. Reproduced with permission from Ref. [82].

Screen printing is a widely used technique for imprinting designs onto a variety of substrates, such as paper, textiles, polymers, glass, or any solid material [82]. This technique involves pushing a viscous paste (or ink) through the orifices of a stencil screen with the help of a rubber blade (known as a squeegee) [83]. The procedure is shown in Figure 10. This method has a long history, with its origins believed to be in China and Egypt [84]. Nowadays, it is a particularly useful method for manufacturing scalable, high-speed printed microelectronics, as it is cost effective, easy to operate, and can be scaled up for larger production.

The key components of the screen-printing process that affect the resulting film are (i) the stencil, (ii) the squeegee, and (iii) the inks or pastes. The stencil is usually made of cotton, natural silk, plastic (nylon or polyester), or woven metal fibers; with plastic and metallic filaments being the preferred materials due to their durability and chemical resistance. The number of openings in the screen per linear inch (mesh count) also affects the quality of the pattern and the film thickness [83]. To create the stencil, a light-sensitive emulsion is applied to the mesh, with the net covering only the non-printable areas [82]. The squeegee is a blade made from a polymer with synthetic materials such as polyvinyl or polyurethane typically used for their sharpness and durability. The squeegee is used to control the spread of paste through the mesh and to adjust the film thickness [82]. The inks or pastes need to have the correct rheology to be able to infiltrate the mesh, and are composed of a solvent or solvent mixture, material particles, and organic binders. The proportions of these components depend on the type of substrate to be imprinted. Commonly used solvents are water and organic solvents, while binders are chosen to match the desired viscosity and functionality [85]. Despite the fact that a considerable number of studies have been conducted on screen printing of electronic materials, the operator must still adjust the deposition parameters and paste formulation in order to achieve the best possible coating. Here, we will look back at several studies related to paste formulation in C-PSCs.

**Figure 11:**
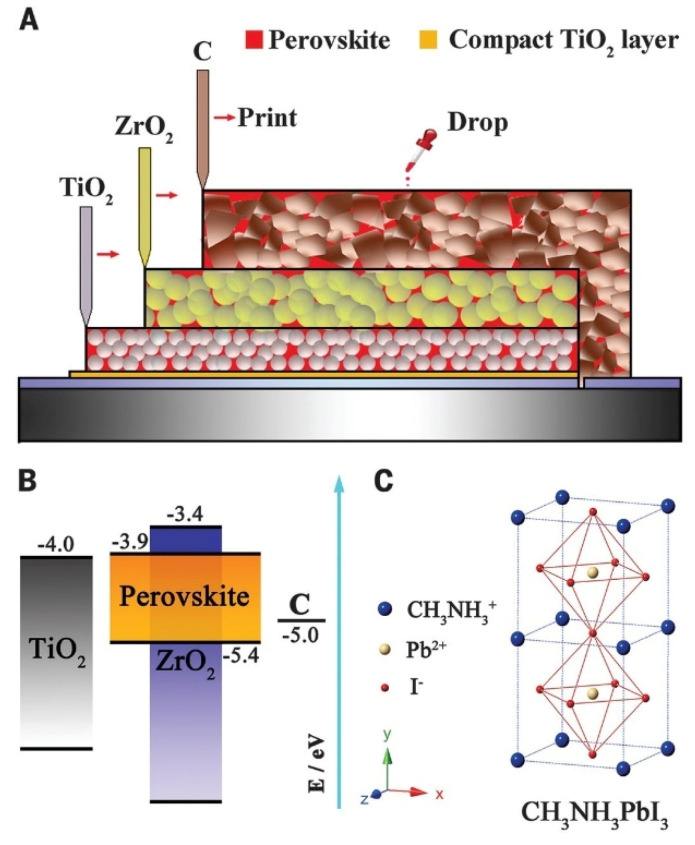
(**A**) Scheme of fully printable triple-stack mesoporous carbon-based perovskite solar cells (PSC), (**B**) Band gap diagram of each layer present in the carbon-based PSC, and (**C**) crystalline structure of MAPbI_3_ perovskite. Reproduced with permission from Ref. [86].

In 2013, Ku et al., reported the first mesoscopic C-PSC using MAPI perovskite (Figure 11) [86]. This triple-stack C-PSC was printed using the screen-printing technique and the layers were composed of TiO_2_, ZrO_2_ or alumina, and carbon. As mentioned before, researchers usually use commercially available pastes such as TiO_2_ paste with particle size around 30 nm [14], and it is further diluted with terpineol in 1:1 to 1:7 ratios to attain the desired thickness. Commercial ZrO_2_ paste is composed of particles with diameters between 20 nm to 40 nm [15] and, depending on the stencil and dilution employed, the layer thickness can be changed from 1.5 μm to 3.0 μm. Carbon paste is also available commercially [15] and, as mentioned before, it consists of graphite and carbon black in different ratios. The carbon layer thickness must be in the range of 8 μm to 20 μm.

The following section is dedicated to homemade paste formulation for the fabrication of triple-stack mesoporous TiO_2_/ZrO_2_/C perovskite solar cells. The section is divided according to the separate layers, where we will show the formulation of each homemade paste in order to enhance the properties of each coating. Most of these formulations are based on the original work from the Han group, in which a corresponding metal oxide (5% to 20%), ethyl cellulose (2.5% to 10% concentration), and terpineol (70% to 92.5%) are mixed to reach an appropriate viscosity. Also, other components such as lauric acid, turpentine alcohol, acetylacetonate, or propylene glycol monomethyl ether acetate and organic binders such as hydroxyethyl cellulose, poly(vinyl pyrrolidone), or polyacrylic resin have been used in the paste formulation. 

#### 2.2.1. Blocking Layer

Spray pyrolysis is typically the method used to deposit the bottom layer (BL) of the triple mesoscopic architecture. However, recently screen printing has also been employed to deposit this layer, demonstrating fully screen-printed C-PSCs and the scalability of this method. Hvojnik and collaborators screen-printed a compact TiO_2_ BL using the commercial Solaronix paste and compared the performance of the screen-printed and spin-coated BLs in C-PSCs [87]. The spin coated BL had the best result, with a PCE of 7.8%. In a recent article, Wang et al. took a different approach, screen-printing the mesoscopic triple-layer from Wonder Solar on top of an In and Al doped compact-TiO_2_ BL [88]. A precursor solution of AVA_x_MA_1-x_PbI_3_ perovskite was then drop-cast on the structure, resulting in a PCE of 15.53% with the doped BL, and 13.49% with a typical c-TiO_2_.

#### 2.2.2. Electron Transport Layer

Research dedicated to this layer is mainly focused in improving the surface area and chemistry of the TiO_2_ film, which results in better infiltration of the perovskite precursor solution, and thus better TiO_2_/perovskite electrical contact. In this sense, Liu et al. developed their own TiO_2_ paste composed of two different NP sizes to enhance the performance of C-PSCs [89]. The TiO_2_ paste was fabricated from a mixture of the oxide powder, ethyl cellulose, and terpineol in a 2:1:7 ratio, respectively. The TiO_2_ powder portion consisted of different mass fractions of 25 nm and 100 nm particles. The perovskite precursor solution was infiltrated into the triple-stack by one-step drop casting. Solar devices with 14.4 wt.% of 100 nm TiO_2_ particles achieved better performance with PCE up to 10.81%; in contrast, those with 25 nm-TiO_2_ particles reached 9.17%. Likewise, Zhao et al. developed a nano-TiO_2_ slurry synthesized from the metal-organic framework (MOF) MIL-125 [90]. TiO_2_ in the anatase phase was obtained by sintering the MIL-125 at 450° C for 4 h. Once the particles were obtained, the slurry was prepared by mixing the powder (5 wt.%) with ethyl cellulose (2.5 wt.%) and terpineol (92.5 wt.%) as binder agent and solvent, respectively, followed by ball milling. Finally, the (MA)_1-x_(5-AVA)_x_PbI_3_ perovskite precursor solution was drop-cast. They obtained devices with PCE up to 13.42%. Furthermore, they synthesized another TiO_2_ powder from the MOF MIL-125-NH_2_, which was employed to obtain a paste containing 4.6 wt.% TiO_2_ anatase, 91.4 wt.% terpineol, 3.3 wt.% ethyl cellulose, and 7 wt.% lauric acid [91]. Fully screen-printed mesoscopic structures were further infiltrated with MAPI precursor solution in DMF. The MIL-125-NH_2_ -based devices achieved a champion PCE up to 12.55%. It is claimed that the cell efficiency enhancement was the result of the large specific surface area, as well as the existence of special pores that made the MOF-derived TiO_2_ layer more favorable to the permeation of the perovskite precursor solution.

In addition to the highly used TiO_2_, BaSnO_3_ coatings have also been considered as ETL since it has a suitable optical band gap of 3.1 eV and high electron mobility of 70 cm^2^/V·s [92]. According to the report, the barium stannate nanomaterial was synthesized by co-precipitation and thermal annealing under nitrogen atmosphere. The paste was fabricated from a mixture of ethanol, turpentine alcohol, acetylacetonate, and ethyl cellulose. Additionally, they obtained a NP dispersion by ultrasound, which was treated by rotary evaporation to obtain the appropriate rheology. The paste was deposited by screen printing, followed by screen printing of the ZrO_2_ and carbon layers. With this triple-stack architecture, the authors achieved PCEs up to 14.77%. The results were ascribed to enhanced charge extraction efficiency and suppressed charge recombination due to the presence of extensive oxygen vacancies inside the particles.

#### 2.2.3. Insulating ZrO_2_ Layer

Liu and co-workers, from the Han group, constructed the triple-stack mesoscopic architecture from the Greatcell m-TiO_2_ paste, diluted with terpineol (3:1.2), along their own previously developed ZrO_2_ and carbon pastes [86]. This scaffold was employed to test the critical thickness of ZrO_2_ insulating layer to achieve an efficient carbon-based mesoscopic perovskite solar cell [93]. The optimal thickness for spacer layer was determined to be 1 μm, with a PCE of 10.30%. Moreover, when the MAPbI_3_ precursor solution was drop-cast at 70 °C (solution and substrate temperature), 12.34% PCE was achieved. The cell improvement is explained by better infiltration at 70 °C, and better quality MAPbI_3_ crystal formation at that temperature. 

**Figure 12:**
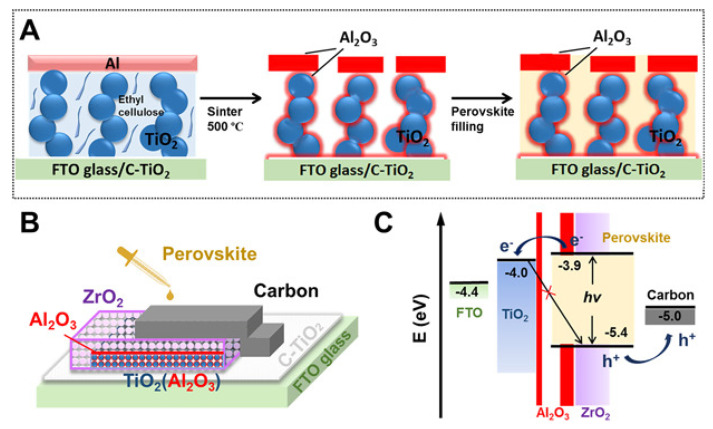
(**A**) Implementation of an extra Al_2_O_3_ layer with m-TiO_2_ and m-ZrO_2_ via a vacuum technique, (**B**) infiltration of perovskite precursor solution through the layers, and (**C**) energy band diagram of hole-conductor-free C-PSC with Al_2_O_3_ interlayer. Reproduced with permission from Ref. [96].

Meng and collaborators fabricated their own pastes of mesoporous TiO_2_, ZrO_2_, Al_2_O_3_, and carbon, to evaluate the substitution of ZrO_2_ isolating layer with Al_2_O_3_, due to its lower cost and better availability [94]. TiO_2_ was prepared from a mixture of 15 wt.% nanosheets of TiO_2_, 6 wt.% ethyl cellulose, and 79 wt.% terpineol. Al_2_O_3_ and ZrO_2_ nanoparticles with diameters of 20 nm and 20–40 nm, respectively, were suspended in a mixture of 9.8 wt.% of the corresponding oxide, 1.98 wt.% of ethyl cellulose, 39.22 wt.% of terpineol, and 49 wt.% of absolute ethanol; this mixture was ball milled during 5 h, followed by rotary evaporation to obtain the appropriate rheology. Carbon paste was prepared according to the procedure developed by Rong et al. [95]. Finally, the (5-AVA)_x_(MA)_1-x_PbI_3_ precursor solution was drop-cast on top of the carbon-based architecture. The zirconia-based spacer solar cells achieved PCE of 8.72%; whereas the alumina-based devices showed an efficiency of 5.48%. These results were explained by the morphology of the films: the larger nanoparticle diameter of the ZrO_2_ material generates bigger pores that allowed better perovskite infiltration. The substitution of the ZrO_2_ scaffold layer with Al_2_O_3_ was further investigated by Xiong and co-workers [96]. The authors prepared their own pastes from recipes previously developed by Han’s group [86]. The mesoscopic TiO_2_ layer was screen-printed using a paste prepared with P25 Degussa powder. To obtain the Al_2_O_3_ layer, they deposited a thin coating of aluminum by vacuum techniques on top of TiO_2_ and sintered it at 500 °C for 40 min (scheme shown in Figure 12). Following, ZrO_2_ and C pastes were screen-printed according to the typical procedure. Infiltration was carried out from a (5-AVA)_x_(MA)_1-x_PbI_3_ precursor solution in γ-butyrolactone (GBL) by drop casting. Solar cells with 10 nm Al thickness and a 1 μm ZrO_2_ layer achieved the best results (PCE of 14.26%) along with a negligible hysteresis effect.

#### 2.2.4. p-Type Mesoporous Metal Oxide: Hole Transporting/Extracting Materials 

**Figure 13:**
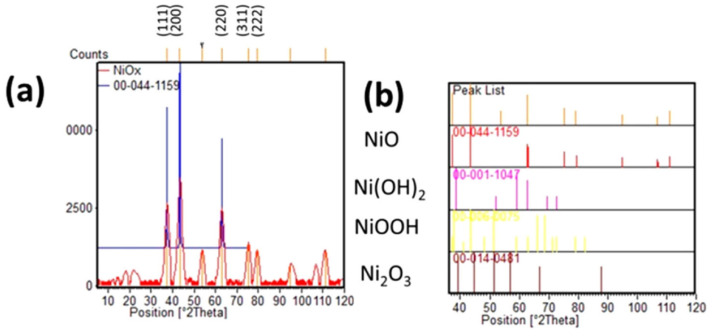
XRD analyses of black NiOx acquired from Sigma-Aldrich: (**a**) powder of NiO, Ni(OH)_2_, NiOOH, and Ni_2_O_3_, and (**b**) mixture of Ni^2+^ and Ni^3+^.Reproduced with permission from [98].

In triple-stack carbon-based solar cells, a low-cost, mesoporous and printable hole transport layer such as nickel oxide can be used between the spacer and carbon layers in order to improve the performance. All studies developed their own NiO paste using approximately the same recipe i.e., 5.0% to 15% of NiO, 3.0% to 7.0% of ethyl cellulose, and 76.0% to 90.0% of terpineol. Another p-type metal oxide that can work as hole extracting layer is Co_2_O_3_ [97]. Behrouznejad et al. developed their own pastes from commercial powders of TiO_2_ (30 nm) and ZrO_2_ (45 nm) from Sigma-Aldrich [98]. The carbon paste is composed of 5 wt.% carbon black, 5 wt.% Al_2_O_3_, 10 wt.% graphite, 20 wt.% ethyl cellulose, and 60 wt.% alpha-terpineol. They used NiO_x_ (30 nm in diameter, from Sigma-Aldrich) paste that worked as a hole extraction layer. When the Al_2_O_3_ layer presented a thickness of 450 nm, which is the critical parameter in this architecture, a PCE up to 12.12% was obtained. The authors conclude that the NiO_x_ layer, with both Ni^3+^ and Ni^2+^ species detected by XRD analyses of the initial nickel oxide (Figure 13), helps to increase the V_OC_ up to 945 mV due to hole hopping from Ni^3+^ sites to Ni^2+^ sites. This layer shifts the hole Fermi-level downward, resulting in an increment in V_OC_. Following this strategy, Tao et al. developed a triple-stack architecture supplemented with a NiO/graphene coating as hole-transporting layer [99]. The authors employed a commercial TiO_2_ paste, and prepared their own Al_2_O_3_, NiO/graphene and C pastes. The Al_2_O_3_ and NiO/graphene slurries were obtained from a mixture of the corresponding oxide powder, ethyl cellulose and terpineol in a 2:1:27 ratio, diluted with ethanol, and ball milled for 10 h. In order to optimize the rheological properties, the ethanol was rotary evaporated. For NiO paste, different amounts of graphene were added and extra terpineol was added to fix the solid contents at 15%. Carbon paste was prepared from a blend of graphite, carbon black, ethyl cellulose, and ZrO_2_ with a 7:2:1:1 ratio, which was ball milled for 10 h. TiO_2_ paste was spin-coated at a speed of 2500 rpm for 30 s, whereas Al_2_O_3_, NiO and carbon layers were screen-printed to reach the stack architecture. The device was finally infiltrated by a two-step process with PbI_2_ in DMF, followed by immersion of the cell into a MAI in IPA solution. The best results were obtained for a NiO/graphene ratio of 100:1 that achieved a device efficiency of 11.72%. Graphene was added in small quantities since its sole purpose was to improve the conductivity and hole extraction ability of nickel oxide layer. Noteworthy, the authors determined that the addition of graphene into the NiO coating made the cell more resistant to humidity, hence it stabilized the cell.

Icli et al. developed a carbon-based mesoscopic substrate using a m-TiO_2_/MgO/NiO/carbon array [100]. They synthesized MgO, TiO_2_ doped with yttrium (Y:TiO_2_) and NiO doped with lithium (Li:NiO) by a methanol combustion method in a flame spray apparatus that guaranteed the incorporation of the Li^+^ and Y^3+^ cations into the corresponding oxide matrix. Then, all oxide pastes were prepared from a mixture of the corresponding oxide powders, ethyl cellulose, and terpineol in a 2:1:10 ratio. The carbon layer was deposited from commercial paste. The architecture was screen printed and infiltrated by a one step drop casting process. The best device performance was obtained for the stacked architecture with 500 nm, 500 nm, and 1 μm thickness for the TiO_2_/MgO/Li:NiO layers, respectively achieving a PCE of 9.63%. In contrast, when the Y:TiO_2_ was used in the stack, the efficiency lowered to just 2.78% due to formation of rutile phase that has worse device performance. Therefore, they concluded that lithium incorporation into the NiO matrix increased the charge collection and transport properties and reduced the overall resistance of the cell.

**Figure 14:**
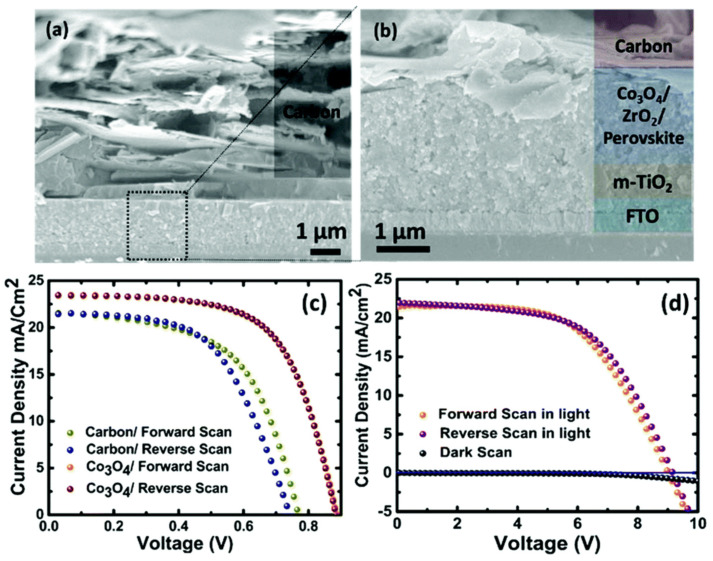
Cross-section of the perovskite/triple-stack mesoporous carbon-based solar cell with Co_2_O_3_ hole-transporting layer: (**a**) low magnification, and (**b**) high magnification, (**c**) J-V characteristics of standard carbon cell with an aperture area of 0.09 cm^2^ (under 1 Sun) without and with Co_3_O_4_ layer, and (**d**) J-V characteristics of module with an active area of 70 cm^2^ and with Co_3_O_4_. Reproduced with permission from Ref. [97].

Bashir et al. employed this methodology to build the triple-stack mesoporous carbon-based scaffold [101]. Between the ZrO_2_ and carbon layers, an 80 nm thin sheet of Cu:NiO_x_/NiO_x_ was inserted as hole conducting layer. The copper oxide paste was prepared by the mixture of Cu:NiO_x_ or NiO_x_ nanoparticles, with ethyl cellulose and terpineol in a 1:10:8 ratio, which was ball-milled, and rotary evaporated to obtain the desired viscosity. The MAPbI_3_ perovskite (5% AVAI) in GBL was infiltrated in one step. Devices of an active area of 0.09 cm^2^ and 0.8 cm^2^ were fabricated, and for a 0.8 cm^2^ cell a record efficiency of 12.79% was obtained; in addition, the cell was stable for up to 6 months without any encapsulation. Using the same approach, the authors also tested a thin layer of Co_2_O_3_ spinel to enhance the hole extraction efficiency in the solar cell [97]. The Co_2_O_3_ paste was the result of a mixture of 10 wt.% oxide, 10 wt.% ethyl cellulose, and 80% terpineol, which was diluted in ethanol, ground by ball milling, and finally rotary evaporated to obtain the appropriate rheological properties to be screen-printed. The initial paste generated layers with 340 nm in thickness, thus different dilutions with terpineol were performed to achieve different layer thickness. The layer resulting from dilution with 1:5 ratio achieved the best results (shown in Figure 14); the same architecture without the spinel layer, achieved PCE of 11.25%.

#### 2.2.5. Carbon Layer

**Figure 15:**
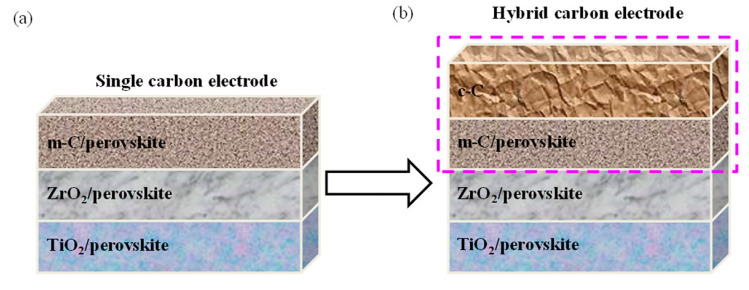
Construction of the: (**a**) single carbon electrode composed of a mesoporous carbon layer, and (**b**) double-layer carbon electrode composed of a mesoporous carbon below a conductor carbon layer. Reproduced with permission from Ref. [105].

As mentioned before, due to inefficient hole extraction and the relatively low open-circuit voltage (V_OC_) of the carbon-based perovskite solar cells, significant efforts are focused on the engineering of the carbon layer. Duan and co-workers modified the carbon layer by the addition of boron-doped graphite, which was synthesized using 5 wt.% boron carbide (B_4_C) with the aim to improve the work function of carbon electrode from 4.81 to 5.10 eV [102]. The paste was prepared from a mixture of boron-doped graphite, carbon black, hydroxypropyl cellulose, ZrO_2_, and terpineol in a 13:4:2:2:60 ratio that was ball milled for 12 h. Then, the typical triple-stack architecture was screen printed and infiltrated with the (5-AVA)_x_(MA)_1-x_PbI_3_ precursor solution by drop casting. With this modification, the device presented an efficiency of up to 13.6%. In contrast, when the carbon layer was fabricated without boron, the resulting cell presented a PCE of up to 12.4%. The improvement in the work function facilitated hole extraction from the perovskite and shortened the PL lifetime, which may be translated into a longer carrier recombination lifetime. Chen et al. further modified the work function of the carbon counter electrode with chloride (C-Cl_x_) to enhance the efficiency of the resulting carbon-based perovskite solar cell [103]. The graphite was modified via reaction of the pristine graphite (XFNano) with hydrochloric acid, washed, and later sodium hypochlorite (NaClO) was added to obtain the C-Cl_x_. The resulting product was used to fabricate the carbon paste from a blend of 1 g carbon black, 3 g of the attained C-Cl_x_ graphite, 0.4 g ZrO_2_ powder, 0.5 g ethyl cellulose and 10 g terpineol. The mixture was ball milled for 12 h. The triple-stack architecture was screen printed and infiltrated with the typical drop casting method. The chlorinated carbon with the C-Cl_0.4_ formula achieved a PCE of 11.75%, which was 1.46 times higher than the pristine device. Chu et al. developed a double carbon layer to improve the conductivity of the electrode [104]. A bottom coating composed of carbon black was first deposited, which was designed to guarantee an intimate contact between the perovskite layer and the electrode. The upper layer, which was a 3:1 mixture of graphite: carbon black, ensured the proper conductivity of the coating. Polyacrylic resin and propylene glycol monomethyl ether acetate were used as binder and solvent, respectively, and ball milling was employed to properly homogenize the paste. With this design, the authors were able to ensure good conductivity with a device efficiency of 14.1%, a V_OC_ of 1.01 V, and excellent stability. Likewise, Jiang a co-workers constructed a double-layer carbon electrode to take advantage of the work function of both layers to enhance the open-circuit voltage and to reduce the series resistance (R_s_) of the carbon-based perovskite solar cell [105]. They prepared a counter electrode with two layers: a high-temperature mesoporous carbon layer (m-C) with high work function and large surface area as a first layer, which was responsible for charge extraction. The second layer was a low-temperature highly conductive carbon layer (c-C), which was responsible for charge collection (see Figure 15). Moreover, they modified another m-C paste by the addition of Co_2_O_3_, CuO, MoO_3_, and NiO p-type oxide powders. The m-C paste was prepared from a mixture of 2 g carbon black (30 nm particle size), 6.5 g graphite, 30 mL terpineol, 1 g ZrO_2_ (20 nm particle size) and 1 g hydroxypropyl cellulose, which was ball-milled for 10 h. Then the p-type m-C paste was obtained by adding different oxide ratios to the former paste. The highly conductive c-C paste was obtained from a blend of 1.5 g carbon black (30 nm particle size), 4.5 g graphite, 10 g terpineol, 1.83 mL titanium (IV) isopropoxide, and 200 μL acetic acid that was ball-milled for 10 h. The champion solar cell achieved 14.15% efficiency using modified m-C paste with 50% NiO as the lower carbon layer. In this structure the ZrO_2_ layer thickness was 3 μm. 

Another work based on the screen-printed double-layer of the carbon electrode is reported by Gong and collaborators. They implemented a carbon black interlayer between the CsPbI_2_Br perovskite layer and the carbon electrode in a planar architecture [106]. The interlayer was fabricated with the mixture of carbon black, ethyl cellulose and terpineol, which was screen-printed with a 325 mesh screen to ensure a proper thickness. With this thin carbon black coating, the authors facilitated the hole extraction due to larger contact area and suitable energy band alignment in the perovskite—carbon black interface. In addition, the research revealed diffusion of carbon black, which resulted in the enhancement of the cell efficiency. Therefore, a PCE of 13.13% was achieved for the inorganic-based PSC with a thin interlayer of 4 μm. In later work, the authors implemented an extra interlayer composed of CuSCN, which afforded a record device efficiency of 15.70% [107].

Hatala et al. tested different compositions of carbon pastes to study its effect on the performance of the carbon-based perovskite solar cell [108]. They changed three parameters: the carbon black concentration, the polymer binder, and the solvents. Graphite powder with particle size < 20 μm, and conductive carbon black with average particle size of 45 nm were used. Ethyl cellulose or poly(vinyl pyrrolidone) as binder agents and diethylene glycol butyl ether, 4-hydroxy-4-methyl-2-pentanone (HMP) and ethyl alcohol as solvents were employed. The pastes were ball milled to achieve a homogeneous mixture. A higher amount of carbon black enhanced viscosity, whereas the lowest sheet resistance was observed with a 3:1 graphite—carbon black ratio. The total carbon amount increased to 35 wt.% resulting in an improvement in conductivity. In addition, a higher concentration of ethyl cellulose increased the viscosity of the paste and enhanced its conductivity. In contrast, a proper ethyl cellulose: poly(vinyl pyrrolidone) mixture resulted in less surface roughness of the layer.

Raptis and collaborators employed aluminium and copper grids integrated into the same double-carbon layer to enhance the conductivity of this coating [109]. They prepared their own low-temperature carbon paste from a mixture of ethyl cellulose binder (12.5%), a mixture of carbon allotropes (29.4%), and terpineol. The triple-stack mesoporous architecture was screen printed, annealed, and finally infiltrated with a (5-AVA)_x_(MA)_1-x_PbI_3_ precursor solution by drop casting. Then, aluminium or copper grids (25 μm thick) were firmly placed on top of the mesoporous carbon layer and the low-temperature carbon layer was deposited to allow the incorporation of the grid into the carbon layer underneath. When a copper grid was used in the carbon-based solar cell, up to 13.15% efficiency was obtained. In contrast, the Al grid resulted in worse performance. In a similar approach, Zhang et al. enhanced the hole extraction efficiency of the carbon electrode via addition of a liquid metal (GaInSn) dopant in the carbon layer, which may also enhance conductivity [110]. The LM-modified carbon paste was fabricated from the mixture of graphite, carbon black, ZrO_2_, and terpineol in a 15:5:4:75 ratio that was ball milled for 24 h. The liquid metal was added in different concentrations of 0.6, 1.2, 2.0, and 3.5% before the milling process. Then the triple-stack mesoscopic architecture was screen printed using the typical method, followed by drop casting of the (5-AVA)_x_(MA)_1-x_PbI_3_ perovskite precursor solution. The modified PSC with 1.2% liquid metal achieved an efficiency up to 13.51% and showed a negligible hysteresis in the J-V curves.

In another study, the effect of different types of carbon black on device performance was investigated [111]. The carbon paste was formulated from a mixture of graphite powder (10 μm flake size), carbon black, ZrO_2_, ethyl cellulose, and terpineol with a 7:1:1:3:23.27 ratio, which was ball-milled for 10 h at 300 rpm. Three types of carbon black, Vulcan carbon (75.1 nm, CABOT), mesoporous carbon (58.0 nm, Sigma-Aldrich) and Super P carbon (47.8 nm, TimCal) were tested, which have different surface area, pore volume, micropore area, and adsorption-desorption pore radius. The best results were achieved for Vulcan carbon, with a device efficiency of 12.55%. The authors conclude that the higher porosity and pore volume in Vulcan black carbon allowed decent wettability of the perovskite precursor solution, excellent infiltration, and strong adhesion within the device stacks. Further, Bogachuk et al. employed different graphite sources in the carbon paste to evaluate the importance of graphite in the sheet resistance of the carbon-based cathode [112]. The authors based their carbon paste formulation on Elcocarb B/SP from Solaronix. They used six different types of natural and synthesized graphite: Flaky (V14, Ito graphite Co., Tado-cho Kuwana-city, Japan), scaly (SPR150, Ito graphite Co., Tado-cho Kuwana-city, Japan), pyrolytic (PC30, Ito graphite Co., Ltd., Tado-cho Kuwana-city, Japan), amorphous (Oriental Industry Co., Ltd., Shenzhen, China), needle graphite (Oriental Industry Co., Ltd., Shenzhen, China), and artificial fine powders (UFG30, Showa Denko K.K., Tokyo, Japan). Among these graphite powders, scaly graphite showed better performance, as it achieved up to 14.28% due to better transport properties and lower series resistance losses. The same carbon paste recipe was used by Tsuji et al. to expand on the investigation with the implementation of extra amorphous graphite [113], which was compared with pyrolytic graphite. Once more, the latter graphite demonstrated better performance with an efficiency of 13.45%. 

Phillips and co-workers developed their own carbon inks, with optimum graphite to carbon black ratio to enhance conductivity and hence performance [114]. They found that the best conductivity, with a resistivity of just 0.029 Ω cm, was obtained with a graphite to carbon black (20–50 nm) ratio of 2.6:1. This mixture was prepared in a 29.4% total carbon concentration and 70.6 wt.% for resin VINNOL 15 wt.% dispersed in 4-hydroxy-4-methylpenan-2-one. Later, the same group expanded their investigation to the effect of photonic flash annealing with subsequent compression rolling on carbon-based inks [115]. The authors prepared three different inks with three different compositions: ink with graphite only, graphite nanoplatelets and the previously developed combination of graphite with carbon black. The ink was formulated as vinyl resin VINNOL (Wacker Chemie AG) 15% in 4-hydroxy-4-methylpentan-2-one. The graphite only inks were formulated in a 22.5 wt.% carbon concentration and 77.5 wt.% dispersed polymer, whereas mixture of graphite and carbon black was prepared with a total carbon concentration of 29.4% (2.6 parts graphite to 1 part carbon black) and 70.6 wt.% resin in solvent. Initial mixing of powders in VINNOL was performed manually, followed by homogenization in a three-roll milling. Photonic annealing used for degradation of the binder and subsequent compression rolling resulted in a reduction in printed film thickness, roughness, and an improvement in particle orientation. These results provided better electrical performance for all printed inks. 

Also, several research groups have obtained carbon materials from biomass and tested them in C-PSCs to analyze the device performance. Mali and collaborators fabricated a carbon hole transporting layer from aloe vera bio-carbon [116]. The biochar was obtained from the aloe vera gel that was carbonized under an argon stream at 1000 °C to ensure proper graphitization. Then, the carbon paste was manufactured simply by ball-milling, mixing the bio-carbon with chlorobenzene in a 1:2 wt.%/vol % ratio. With this simple manufacture, the resulting paste was appropriate for screen-printing on top of a typical m-TiO_2_/m-ZrO_2_ stack. The substrate was infiltrated by drop casting with a solution of MAI and PbI_2_ in γ-butyrolactone. This device presented an efficiency of up to 12.58%. For comparison, they also fabricated spiro-MeOTAD/Au-based spin-coated PSCs, which achieved only 3.2% higher PCE than carbon biomass-based C-PSC.

### 2.3. Inkjet Printing

**Figure 16:**
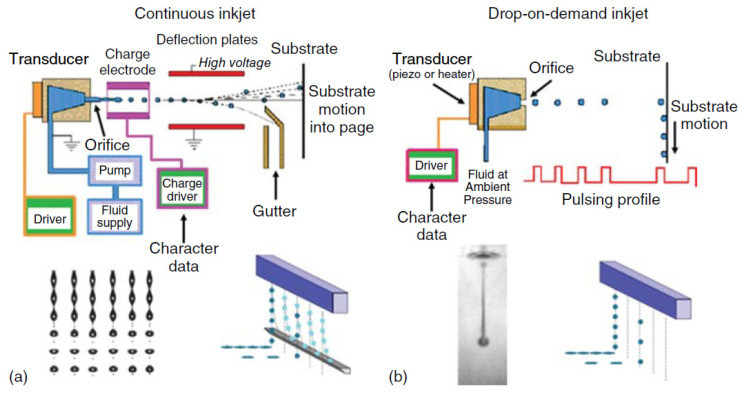
Working principles of inkjet printers: (**a**) CIJ; and (**b**) DOD. Reproduced with permission from Ref [124].

Inkjet printing technology has been widely used in photovoltaic device fabrication since it allows to print the active materials on the solar cell stack [117]. Inkjet printing provides flexibility in thickness and shape, and it is considered a material-efficient technology. The key requirements for ink materials are low viscosity, fine particle size if the ink is a colloid or a suspension, and low volatility to avoid nozzle clogging [118,119]. Inkjet printing can be divided into two modes of operation, continuous (CIJ) and Drop-on-Demand (DOD). In a CIJ printer, charged droplets are controlled by an electrostatic field during the printing process. The imaging droplets pass through to the substrate while other droplets are deflected to an ink catcher for re-use (Figure 14a). A DOD printer ejects ink droplets when a pulse of voltage is applied, without any drop deflecting and catching. Generally speaking, the CIJ printer has a higher jetting frequency, and the DOD printer has a much simpler inkjet head structure [118,120]. Their working principles are schematically shown in Figure 16. There are two principal mechanisms of propelling ink drops in an inkjet printer head, piezoelectric propelling, and thermal bubble propelling. In the piezoelectric inkjet head, a voltage is applied to the piezoelectric pressure transducer to make it bend or change shape, which repels the ink out of the nozzle, as schematically shown in Figure 14b. Drop on demand inkjet printing can be used for the fabrication of C-PSCs by printing all the oxide layers in the stack as well as the organo-metal halide absorber [121,122,123].

In 2007, the first organic solar cell fully fabricated by inkjet printing technique was reported with low efficiencies from 3 to 4% [125,126,127]. In 2014, researchers started using inkjet printing technology to print the functional materials to fabricate PSCs [128,129,130]. Even though studies on inkjet-printed PSCs are few, substantial achievements have been reported [131,132]. Efficiencies higher than 21% have been recently reported for conventional metal electrode devices [133]. Here, we provide several examples of inkjet-printed C-PSCs and the effect of the ink or printing parameters on the device performance. In 2016, Hashmi et al. reported infiltration of the perovskite precursor solution using inkjet printing in the HTM-free carbon counter electrode-based PSCs [134]. The inkjet infiltration of the perovskite precursor solution was performed for a triple-stack (TiO_2_/ZrO_2_/Carbon) mesoporous substrate using the perovskite precursor ink containing 5-AVAI. They used 5-AVAI because it significantly slows down perovskite crystal growth before and after the deposition of the precursor ink, thus preventing the inkjet printer cartridge from clogging, and also provides an opportunity for precise patterning and controlled volume dispensing of precursor ink. The fabricated devices had an efficiency of 8.1% when infiltrated with perovskite precursor in an area of 0.16 cm^2^ using an inkjet printer, and after three weeks of storage in the dark under vacuum showed a high stability and a performance increase to 9.53%.

**Figure 17:**
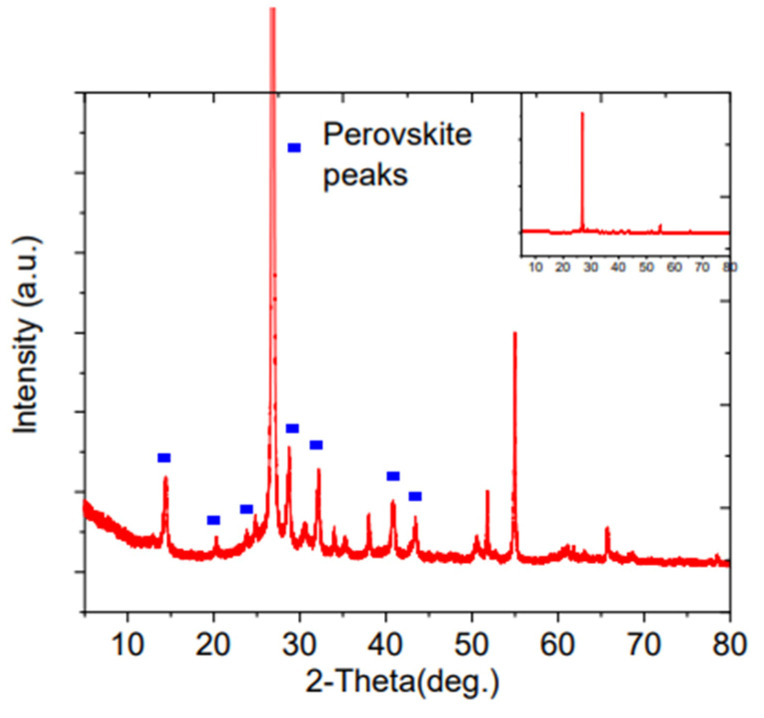
X-ray diffraction (XRD) patterns after infiltrating of CH_3_NH_3_PbI_3_ (MAPI) and annealing. The dominating (101) reflection at 2θ = 25.3° is attributed to anatase TiO_2_ (inset). At reduced intensity scale, the characteristic reflections of the cubic phase of the MAPI perovskite crystal are clearly visible (blue squares). The absence of a reflection peak at 2θ = 12.5° reveals that all the precursor ink has been transformed to the hybrid metal halide perovskite and that no un-desirable PbI_2_ is present. Reproduced with permission from Ref. [135].

In 2020, Verma et al. achieved inkjet-printed carbon-based solar cells with four layers out of five, i.e., c-TiO_2_, m-TiO_2_, and m-ZrO_2_, as well as the perovskite precursor ink, using environmentally friendly non-halogenated solvents [135]. For the c-TiO_2_, they formulated an ink from titanium diisopropoxide bis(acetylacetonate) (TAA) using an appropriate dilution with binary mixtures such as ethylene glycol: iso-propanol (IPA), tetralin: lPA, terpineol: IPA, ethyleneglycol: ethanol, tetralin: ethanol, and terpineol: ethanol. The TAA/terpineol: xylene mixture 1:16 vol/vol yielded stable jetting as well as very homogeneous wet and dry film formation. The final ink composition had a viscosity of 2.1 mPas and a surface tension of 23.1 mN/m. The m-TiO_2_ ink was developed by diluting the commercial screen printing paste (Solaronix) into a terpineol: IPA mixture. For the m-ZrO_2_, the Solaronix paste was diluted with a binary solvent. The ink with 6 vol of screen-printing paste in 5:5 (vol:vol) of binary solvents gave stable jetting with no satellite formation as well as homogenous layer formation. The oxide layers and the carbon electrode were first sintered at elevated temperature and then infiltrated with the standard MAPI by inkjet printing at room temperature. After infiltration and drying, the devices were post-treated in damp heat for 100 h. As shown in Figure 17, this treatment increases the crystallinity of the perovskite layer. They obtained solar cell devices of an active area of 1.5 cm^2^ with an efficiency of 9.1%. 

In 2021, Karavioti et al. provided a direct comparison between spin-coating and inkjet-printing techniques for the fabrication of fully ambient air-processed perovskite absorbent layers for C-based HTL-free PSCs [136]. The results showed that the inkjet-printed perovskite layer presented a discontinuous morphology due to a “coffee-ring” effect unlike in the case of spin-coating, where a uniform morphology was obtained. This is mainly because in inkjet-printing, the applied perovskite precursor solutions should be of a much higher concentration compared to the corresponding solutions used for spin coating. In inkjet printing, the solvent evaporation rate is lower than in the case of spin coating, leading to a poor crystal structure, whereas in the case of spin-coating most of the solvent is quickly removed through centrifugal forces. Another reason is related to the wettability of the substrate by the ink, in particular, over-wetting. Moreover, the FTO/c-TiO_2_/m-TiO_2_/perovskite system fabricated by inkjet printing presented a lower light-harvesting efficiency (LHE) compared to cells fabricated by spin coating. These differences had an impact on the electrical characteristics of the solar cells. The devices fabricated by the inkjet printing technique achieved an efficiency of 8.40% compared to 10% for the solar cells fabricated by spin coating.

Chalkias et al. devised a strategy based on the jet-ability of the ink and on the wettability of the substrate with the ink to reduce “coffee ring defects” that appeared during ambient air inkjet printing processing of the PSCs [137]. With different concentrations of the perovskite precursor ink (up to the limits determined by the solubility of the solutes in the solvent used), better charge transportation and recombination kinetics and an improvement of the external and internal quantum efficiency of the developed solar cells were achieved. C-based HTL-free PSCs using the optimized ink (1.8 M of perovskite precursor) reached an efficiency >12% for a cell with an active area of 0.34 cm^2^, which is among the highest reported values for the architecture c-TiO_2_, m-TiO_2_ and perovskite layers deposited by inkjet printing. Finally, by the combination of inkjet printing and screen printing perovskite sub-modules of 34.2 cm^2^ with an average efficiency value of 9.09% were developed to demonstrate the scalability of the technology. While the ink properties play an essential role in determining the solar cell performance, the fluid dynamics calculation of ink is crucial. However, inkjet printing of carbon and metal oxides for electrodes remains a challenge and requires further ink and process development.

### 2.4. Slot-Die Coating

**Figure 18:**
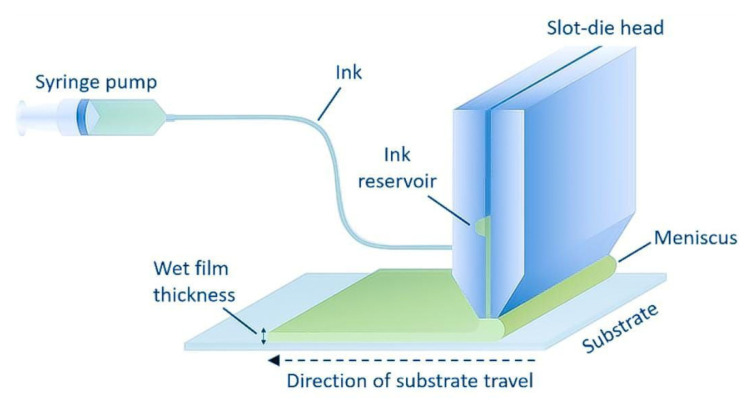
Schematic illustration of the main components in a slot-die processing set-up.

Slot-die coating is an attractive, low-cost printing technique that is used for the deposition of the perovskite and other layers because of its high uniformity in large-scale production and thickness control over a broad range. Nevertheless, slot die coating has not been considered in the literature as a promising coating technique for C-PSCs [138]. As illustrated in Figure 18, a standard slot-die coating process includes an ink reservoir and slot-die head positioned towards the substrate, then the ink is pumped into the head using a syringe pump, with the ink forced out of a narrow slit along the length of the coating head. The ink then creates a liquid bridge between the substrate and slot-die head, and deposition happens upon moving the substrate across the coating head [138]. Slot-die coating covers inks within a wide viscosity window ranging from 1 to 10,000 mPa s, and final dry thickness from a few nanometers to tens of microns [139]. In this technique, the determination of the operational limits to set parameters such as coating speed, flow rate and coating gap must be selected carefully to avoid coating defects [140].

Khambunkoed et al. fabricated carbon-based methylammonium-free PSCs in 2021, utilizing amorphous zinc tin oxide (ZTO) as the ETL [141]. The device was produced with a homemade slot-die system, and the inks used were prepared from commercial materials. Amorphous ZTO was chosen due to its beneficial optical and electronic properties, such as high electrical conductivity, high electron mobility, and high transparency. The device had an optimum PCE of 9.92% when the ZTO thickness was around 48 nm. This PCE value was comparable to that of a PSC with spin-coated ZTO. The short circuit current density, open circuit voltage, and fill factor of the device were 18.86 mA/cm^2^, 0.94 V, and 56%, respectively.

### 2.5. Blade Coating

**Figure 19:**
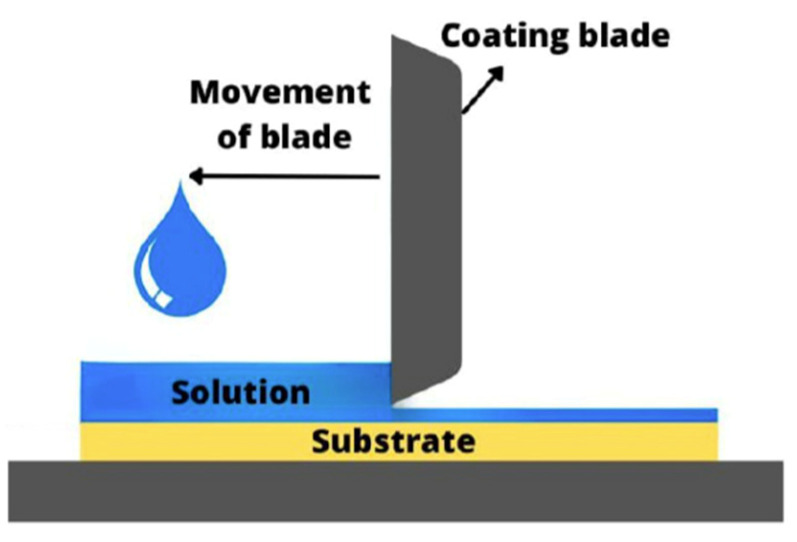
Schematic illustration of the “doctor blade” film deposition process.

Doctor blade coating is one of the widely used techniques to produce thin films on large area surfaces. The operation principle ensembles the screen-printing approach. According to Figure 19, in the doctor blade process, the precursor solution is applied between the coating head and the substrate; then, the substrate is coated by moving the blade or knife across the substrate and the solvent is evaporated through heat treatment to obtain the film [142]. Furthermore, the controllable substrate temperature can impact the quality of the film. The inks/pastes used in these processes usually require large amounts of binders and thickeners to produce the high viscosities (1000–10,000 mPa s) required for reproducible and reliable production of films. Viscosities can be increased with the addition of polymeric additives such as glycerol or ethylene glycol or ethyl cellulose [143]. Coating thickness and speed play a vital role in determining interfaces quality. This technique is a relatively simple and scalable fabrication method for C-PSCs [144].

**Figure 20:**
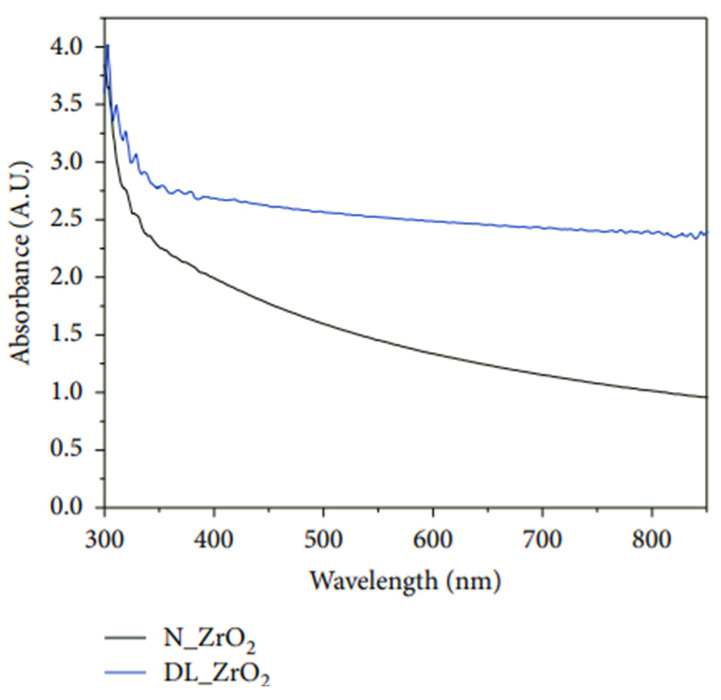
UV-Vis absorbance spectra of ZrO_2_ film (N_ZrO_2_) and double-layered film (DL_ZrO_2_) deposited on glass. Reproduced with permission from Ref. [145].

Syrrokostas et al. reported on the fabrication of C-PSCs with double-layered ZrO_2_ films using the doctor blade method [145]. This included the use of commercial zirconia nanoparticles of both nano and micro sizes, which acted as spacer and light scattering material to increase the light harvesting efficiency and yield an enhanced photocurrent density. The UV-Vis absorption spectra of the N_ZrO_2_ and DL_ZrO_2_ films (see Figure 20) reveal an increased absorbance, covering all the visible and the near-infrared region, from 400 nm to 800 nm, in the second case. The increased absorbance for the DL_ZrO_2_ film is ascribed to the higher scattering ability owing to the size of the particles. The presence of the large ZrO_2_ particles in the double layered spacer film results in an increase of the optical path length, due to the improved scattering efficiency; this resulted in a 30% improvement in efficiency. 

Tian et al. reported further advances by producing C-PSCs using commercial materials and incorporating fullerene C60 as the ETL, in addition to the modification of the ITO substrate with a self-assembled monolayer of 3-aminopropyl triethoxysilane (APTES) [146]. This combination enabled interfacial charge extraction, ultimately resulting in reduced hysteresis in the J-V curve. They report a remarkable PCE of 18.64%, one of the highest reported for C-PSCs, which was found to be stable for over 3000 h. Bidikoudi et al. explored the use of different ammonium iodide (AI) precursors as additive and post-treatment agent in the multiple-cation mixed halide perovskite precursor solution, which enabled the deposition of homogeneous films of ZrO_2_ and carbon with commercial materials [147]. This led to an increase in power conversion efficiency by 33%, with further post-treatment with AI and ethyl-AI raising the efficiencies to 9.77% and 9.15%, respectively. The authors highlighted the importance of selecting the right materials for each part of the solar cell and the substantial influence of the precursor and post-treatment solutions in the C-PSCs fabrication process. 

### 2.6. Spray Coating

**Figure 21:**
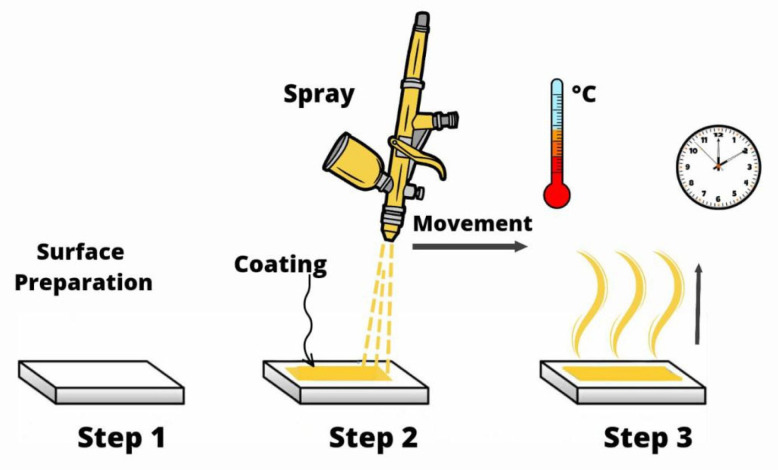
Schematic illustration of the spray coating deposition process.

Spray coating is an effective technique to produce ETL, HTL and other films, as well as the perovskite layer, on various kinds of substrates [130,148]. It is recognized as an attractive method for fabricating large-area, high-throughput, and low-cost PSCs and modules. The spray coating technique can be classified into different methods, including ultrasonic spraying, pneumatic spraying and electrospraying. The classification is based on the droplet dispersion over the substrate [149]. Figure 21 represents the spray coating method. During the spray deposition process, the hot substrate comes in contact with the precursor solution droplets; this leads to heating up and, consequently, drying, yielding faster and uniform crystallization and in turn, better surface morphology [150].

In 2017, Yang et al. reported a simple ultrasound spray deposition method to prepare pure MWCNT films to improve the contact at the perovskite/carbon interface [151]; the c-TiO, m-TiO_2_ and PbI_2_ layers were deposited on FTO/glass by spin coating. Commercial MWCNTs were dispersed in chlorobenzene to form a homogeneous ink, and the MWCNT layer was deposited onto the perovskite layer using a home-made compressed air gun-based spray system. The PCE of the cell with an active area of 0.08 cm^2^ was 14.07%. Moreover, they deposited a sub-monolayer of NiO nanoparticles prior to the MWCNT deposition to enhance the hole extraction efficiency and reduce the recombination rate obtaining up to 15.80% of efficiency, which is among the highest efficiency reported for C-PSCs. 

Another interesting report was published by Wu et al. on the use of electrospray (ES) coating to fabricate C-PSCSs [152]. This technique utilizes a high voltage to atomize a flowing solution into charged micro-droplets and can be used to deposit any layer in the perovskite solar cells. ES is distinguished from other techniques due to the advantages of versatility, scalability, designability, and low materials waste [153]. Wu et al. developed an electrospray deposition technique for continuously printing C-based HTL-free PSCs. All the layers, i.e., TiO_2,_ perovskite and carbon were continuously printed through ES in the ambient atmosphere at low temperature. Commercial precursor solution, paste and solvents were used to prepare the dispersions. The fully ES printed carbon-based PSCs showed V_OC_ of 1.03 V, J_SC_ of 24.35 mA cm^−2^, FF of 57.7% and PCE of 14.41%. Even though this technology is not widely used for perovskite fabrication, the electrospray-assisted technique is promising for C-PSCs commercial manufacture.

## 3. Scale-Up

Although the PCE of PSCs is high enough in small areas, it is necessary to fabricate large-scale perovskite solar modules for future commercialization of this PV technology. However, scientific challenges, technical and techno-economical issues are magnified while increasing the substrate size for the fabrication of larger devices. For a successful scale-up, novel abundant and non-toxic materials, new device architectures, easy and low-cost fabrication processes, and adequate choice of printing technique compatible with the ink formulations and substrate size are required. In addition, in a production line of large-scale devices, all manufacturing steps should be compatible and match with a continuous flow process to avoid a slow production rate and, hence, higher manufacturing cost [154,155]

One example of a conflicting fabrication technique with large-scale substrates is spin coating. Unfortunately, spin coating technique works well for small devices with less than 1 cm^2^. Another problem that is magnified when up scaling the perovskite PV technology is lead toxicity. Still, lead-based perovskite solar cells possess by far the highest efficiencies. To overcome this issue, investigation on lead-free perovskite PVs and effective encapsulation of the entire device should be the important subjects of future research. As mentioned before, the unstable metal top electrode and degradation of perovskite material, which are the main reasons of device instability, have motivated researchers to find stable top electrode alternatives, such as carbon, and new perovskite formulations. Han et al. demonstrated that the carbon-based perovskite solar cell as a fully printable architecture is a promising candidate for large-scale applications related to the easy and cost-effective manufacturing process using stable and abundant materials [86]. Although the carbon-based perovskite devices have shown potential for scale-up, one barrier for large-scale application is the multiple sintering processes required in their manufacture. The sintering process helps to eliminate all organic binders from the printing pastes and results in a better interconnection between nanoparticles. However, it is costly and time-consuming, which reduces the production throughput. One solution is developing low-temperature processing materials such as aqueous-based inks and low-temperature carbon pastes. Unfortunately, the efficiency of low-temperature-based PSCs is still low. This could be due to the adhesion problems of low-temperature and aqueous-based inks to the substrate and poor particle interconnections. There are different deposition methods, including slot-die coating, screen printing, spray coating, roll-to-roll printing that can be used for the fabrication of large area C-PSCs. Rong et al. compared different fabrication methods for large-scale fabrication of PSCs and suggested that among all these methods, screen printing and slot-die coating would be the most promising methods [156]. In the following section we present several examples of large-area C-PSCs fabricated by different printing techniques. 

### 3.1. Screen-Printed Modules

Screen printing is the most employed method to build modules and mini-modules because of the easy scalability of this technique. Priyadarshi et al. first fabricated mini-modules of 70 cm^2^ of active area using the screen printing technique [157]. The device was fabricated employing the commercial pastes for compact TiO_2_ (Greatcell), mesoporous TiO_2_ (Greatcell), mesoporous ZrO_2_ (Solaronix) and two homemade carbon pastes consisting of graphite and carbon particles of less than 500 nm in size. The two carbon pastes had different amounts of ethylcellulose as binder agent. They found that lower concentration of binder enhanced the conductivity of the layer. A third commercial carbon paste (Greatcell) was used to compare with the homemade pastes. The best result was a PCE of 10.03% corresponding to the commercial carbon. Later, the same group improved the previously fabricated mini-modules by inserting an 80 nm thin sheet of Cu:NiO_x_/NiO_x_ as hole conducting layer between ZrO_2_ and carbon layers [101]. The MAPbI_3_ perovskite precursor with 5% 5-AVAI in GBL solvent was infiltrated in one step. Large-area devices with 70 cm^2^ of active area were fabricated reaching stable performance for up to 6 months without any encapsulation. For these large-area devices the precursor solution of MAPbI_3_ perovskite (3% 5-AVAI) was deposited in one step by slot-die coating with a width of 200 mm for the slot die head. In contrast, when a thin layer of spinel Co_2_O_3_ was used in these mini-modules, a device efficiency of 11.39% was obtained [97]. 

**Figure 22:**
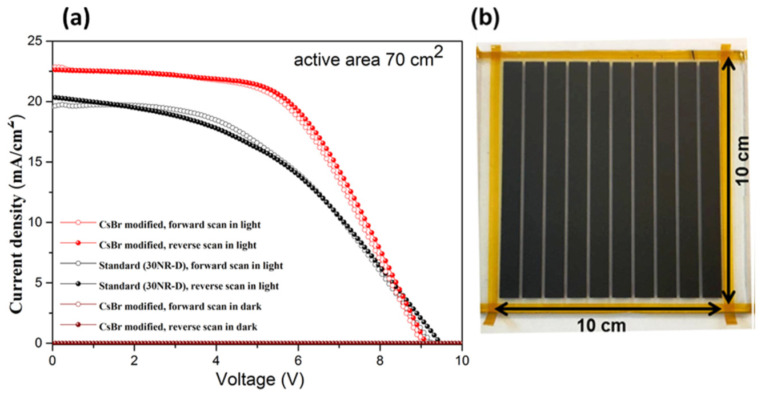
(**a**) Performance of 70 cm^2^ mini-module with CsBr-modified TiO_2_ layer in comparison with a standard carbon-based perovskite solar cell; (**b**) Image of a fully printable mini-module. Reproduced with permission from Ref. [4].

In a recent paper, the group further improved the performance of the 70 cm^2^ mini modules [4]. They modified the formulation of the TiO_2_ commercial paste HPX-100 (Cristal) with variable amounts (from 4.0% to 5.0%) of CsI, CsBr, and CsCl. To compare the efficiency of the homemade pastes, the authors tested the commercially available HPX100CsI and HPX100CsBr, which are modified with CsI and CsBr, respectively, and the typical commercial paste from Greatcell. A PCE of up to 11.55% was obtained for the TiO_2_ paste modified with 4% CsBr. The resulting mini-modules had negligible J-V curve hysteresis, good reproducibility (Figure 22), and excellent environmental stability. 

Hu and co-workers [8] constructed modules with an area of 10 × 10 cm^2^ from the pastes initially developed by the Han group [10,86]. These modules possess an active area of 49 cm^2^ and were infiltrated with a (5-AVA)_x_(MA)_1-x_PbI_3_ perovskite precursor solution in γ-butyrolactone. When the modules were composed of 10 sub-cells, the device reached a PCE up of 10.4%, V_OC_ of 9.3 V, J_SC_ of 2.0 mA/cm^2^, and FF of 0.56, with outdoor stability of 1 month and shelf-life stability of >1 year. Grancini et al. further expanded the stability of the 10 × 10 cm^2^ modules to >10,000 h under continuous irradiation with zero loss in performance measured under controlled standard conditions ^9^. The fabricated modules had an active area of 46.7 cm^2^ and were infiltrated with the same 3D/2D perovskite precursor solution in GVL. These modules reached an efficiency of 11.164%, V_OC_ of 7.05 V, J_SC_ of 2.247 mA/cm^2^, and FF of 0.704.

Raptis and co-workers employed copper and aluminium grids to enhance the conductivity of the carbon electrode in mini-modules of 11.7 cm^2^ [109]. The authors employed commercial TiO_2_ paste from Greatcell, ZrO_2_ paste from Solaronix, and carbon paste from Gwent electronic materials, to fabricate the typical triple-stack mesoporous architecture. The substrates were infiltrated with (5-AVA)_x_(MA)_1-x_PbI_3_ precursor solution by drop casting. Then, they prepared a low-temperature carbon ink that was used to secure the copper or aluminium grid (25 μm thickness). This carbon ink was a mixture of ethyl cellulose binder (12.5%), carbon allotropes (29.4%), 1-butanol, and terpineol. With this architecture, they obtained a PCE of 11.05%, V_OC_ of 5.11 V, I_SC_ of 42.73 mA, and FF of 59.18%, for copper grid reinforced mini modules. In comparison, when a device was evaluated before implementation of copper grid, a PCE of 7.70% (V_OC_ of 5.02 V, I_SC_ of 41.35 mA, and FF of 43.44%) was obtained. 

Lee and collaborators further developed mini-modules with an active area of 9.75 cm^2^ under indoor conditions [158]. When an LED light of 1000 lux was used to test the device, the device achieved a P_max_ of 683 μW, V_OC_ of 3.60 V, I_SC_ of 269 μA, and FF of 70.5%. In a recent article, the group up-scaled the mini-module area to 220 cm^2^ [159]. The modules were infiltrated with a green precursor solution of (5-AVA)_x_(MA)_1-x_PbI_3_ perovskite in GVL:methanol in a 9:1 volume ratio. Outstandingly, the module achieved a PCE up to 9.05%, V_OC_ of 18.4 V, J_SC_ of 21.34 mA/cm^2^, and FF of 51.0% in forward scan.

Bogachuk and co-workers employed commercial pastes to manufacture mini-modules of 10 × 10 cm^2^ geometrical area, and 56.8 cm^2^ active area [160]. These devices, with initial conversion efficiency of 11.1%, were tested using the “hotspot test” implemented in the IEC 61215–2:2016 norm to evaluate the stability of solar cells. These modules were tested for 1 h at 50 °C, and no visible signs of degradation were observed. Noteworthy, the performance of the devices slightly improved (OCE: 12.1%) after 1 h of hotspot test. 

### 3.2. Other Printing Techniques

In regard to other printing techniques, fewer articles have been devoted to scaling up C-PSCs. Most of the larger area devices were created using a combination of screen printing and other methods to print or infiltrate one part of the PV architecture. Duong et al. revealed a blade coating procedure to examine the effect on the morphology and photoluminescence properties of perovskite films via a spray-assisted deposition technique using a multistep spin–spray [161]. Initially, c-TiO_2_ was spun coated onto the FTO substrates from titanium diisopropoxide bis(acetylacetonate) precursor solution. Subsequently, m-TiO_2_ layer was applied on the TiO_2_ BL/FTO substrate by spin coating utilizing TiO_2_ commercial paste diluted in methanol. Then, solutions of PbI_2_ in N,N-dimethylformamide (DMF) were spin-coated on the top of the m-TiO_2_, followed by the incorporation of MAI/IPA into the PbI_2_ layer using an air gun at various substrate temperatures and spray speeds. Lastly, a conductive carbon-coated copper foil was attached directly to the surface of the perovskite layer. This led to a void-free MAPI bilayer with a large grain size, which reduces moisture absorption at the grain boundaries, delays degradation of the organic/inorganic components, and boosts carrier extraction in the fabrication of PSCs. Solar devices with an area of 2.25 cm^2^ exhibited a maximum PCE of 10.58%.

Haruta et al. proposed a one-step blade coating method for the deposition of CsPbBr_3_ thin films using the mist deposition method to ensure efficient carrier transport [162]. The precursor solution was created by mixing CsPbBr_3_ powder with DMSO and DMF. DMF was added to reduce the solvent viscosity and thus enable mist generation. This CsPbBr_3_-based device exhibited remarkable PV performance (PCE of 8.3%), which is the highest for a CsPbBr_3_-based PSC fabricated through a one-step solution process and is comparable to those devices made by two-step solution processes.

Inkjet-printed PSCs are susceptible to “coffee ring” defects, however Chalkias et al. proposed a strategy to reduce their appearance during ambient air inkjet printing processing [137]. By changing the concentration of the perovskite precursor ink, better charge transportation and recombination kinetics were achieved, leading to improved external and internal quantum efficiency. Combining inkjet printing and screen printing, the authors developed HTL-free C-PSCs sub-modules with an average efficiency of 9.09%, covering an area of 34.2 cm^2^, which demonstrates the scalability of this technology. Small-area devices, using the optimized perovskite precursor ink, exhibited an efficiency of more than 12% with 0.34 cm^2^ of active area; this is one of the highest reported values for the inkjet-printed c-TiO_2_/m-TiO_2_/perovskite/carbon architecture.

Verma et al. fabricated C-PSCs using the slot-die technique, depositing a perovskite precursor solution on the stripes of c-TiO_2_, m-TiO_2_, m-ZrO_2_, and carbon in a substrate area of 100 cm^2^ and active area of 1.5 cm^2^ for each stripe [163]. To develop the ink rheology for each layer, they initially utilized commercial materials, diluting the inks for slot-die coating with a lower volume fraction of binders. For the c-TiO_2_ ink, they combined a low boiling point and high boiling point solvent to produce a homogenous film, whereas for the m-TiO_2_, m-ZrO_2_, and carbon inks, alcohol solvents such as *i*-PrOH and EtOH were used. The efficiency of the best performing cells was over 12%, comparable to the values reported for cells manufactured by screen printing. Additionally, they demonstrated the shelf-life stability of fully slot-die coated cells exceeding 1 year under ambient conditions.

## 4. Summary and Future Scope

Carbon-based perovskite solar cells can be fabricated from low-cost and easy printing techniques. For each technique, printing parameters must be optimized to obtain highly efficient devices. In addition to the printing parameters, ink formulation and properties play a crucial role in the quality of deposited layers. Table 1 compares the printing parameters, ink properties, and the highest reported efficiency of the C-PSC device. 

Although carbon materials have been identified as an alternative to metal electrodes in order to address some of the drawbacks of metal top contacts in perovskite solar cells (PSCs), such as instability and high cost, a significant obstacle to their commercialization is the low device performance due to the poor adhesion of the carbon with the lower layers and inadequate contact with the perovskite phase, leading to significant charge recombination. This is attributed to the morphology of the perovskite layer and the inherent physical properties of carbon materials, such as crystal structure, size, and electrical conductivity. Therefore, for C-PSCs to become a mature technology for commercialization, device stability (lifetime), processing cost, and safety are essential criteria that must be taken into account. Various strategies have been proposed to improve the performance and stability of C-PSCs through interface engineering between the perovskite and carbon layers, such as by: (i) inserting an interlayer to improve the carbon work function; (ii) reducing the energy level mismatch and increasing the contact area; and (iii) modifying the perovskite precursor composition using antisolvent including carbon nanoparticles during perovskite deposition to eliminate the pores between the perovskite and carbon top electrode. Using these strategies, the fabrication of C-PSCs with excellent power conversion efficiency has been achieved. Low-temperature C-PSCs are also being investigated, as they offer advantages in terms of process cost since curing temperatures lower than the perovskite decomposition temperature are needed, thus eliminating the need for infiltrating the precursor solution through a thick stack of carbon and spacer layers such as ZrO_2_ or Al_2_O_3_. 

Concerning process safety and non-toxicity, development of lead-free C-PSCs as well as incorporation of green solvents, such as GVL-based precursor solutions, are attractive strategies to be further investigated. Also, recently the utilization of biomass such as aloe vera plant, bamboo chopsticks, peanut shell, phragmites australis and corn stalk bio-carbons has grabbed the attention because of the simple, inexpensive synthesis routes of these materials. 

Although device stability is one of the important advantages generally reported for the C-PSC technology, up to now, the research community has been focused mostly on efficiency improvement. This is necessary since this configuration is quite recent, and more time is needed to fully examine the device lifetime at longer periods of time (several years). In addition to the device performance, C-PSC manufacturing cost and toxicity must be the subject of investigations in order to increase the technological readiness level and the potential of this technology for commercialization. 

## Figures and Tables

**Table 1:** Printing parameters, ink properties and the highest reported efficiency of the C-PSCs [125,140,164,165,166,167,168,169,170,171,172,173,174,175,176,177,178].

(1) Method	(2) Printing Parameters	(3) Layers	Ink Properties			Rate of Ink Usage (U) & Waste (W)	(4) Film Thickness Range (µm)	Printing Speed (m/min)	Throughput (m^2^/min)	Scalability	Structure	The Highest Reported PCE (%)/Active Area (cm^2^)	Ref.
			Viscosity (Pa.s)	Surface Tension (mN/m)	Max Particle Size (nm)								
Spin coating	spinning mode (dynamic, static), spin speed and time, and acceleration/deceleration rate, drop volume	ETL, HTL, Perovskite	Low	Up to 25	Up to 50	U: low, W: very high	0.1–0.5	Very low-batch size	Very low -batch size	No	BL-TiO_2_ (spin coating)/m-TiO_2_ (spin coating)/MAPI (spin coating)/C (blade coating)	17.42/0.1	[34]
											BL-TiO_2_ (spin-coating)/m-TiO_2_ (spin-coating)/ZrO_2_ (spin-coating)/MAPbI_3_: EACQDs as antisolvent (spin-coating)/B-MWNTs (drop casting)/carbon (screen printed)	15.14/0.06	[80]
											BL-TiO_2_ (spray pyrolysis)/m-TiO_2_ (spin coating)/Al_2_O_3_ (spin-coating)/MAPbI_3_ (PbI_2_ embedded with B-MWNTs, spin-coating)/boron (drop casting)	15.23/No data	[78]
Screen printing	mesh and squeegee type, mesh tension, distance between the screen and substrate, angle between the squeegee and screen, pressure and speed of the squeegee, amount of loaded paste	ETL, HTL, Perovskite, Carbon	Moderate-High 2.5- 5	Up to 50	20–20000 (1/10th of mesh opening)	U: high, W: low	0.5–20	Flatbed: up to 35 Rotary: up to 100	Up to 70	Yes	BL-TiO_2_ (spray pyrolysis)/BaSnO_3_ (homemade)/m-ZrO_2_/C (all screen- printed)/PbI_2_.MAI, MACl (drop casting)	14.77/0.1	[92]
											BL-TiO_2_ (spray pyrolysis)/m-TiO_2_/Al_2_O_3_ (homemade)/NiO (homemade)/C (all screen-printed)/MAPbI_3_ (drop casting)	15.03/0.7	[178]
											BL-TiO_2_/m-TiO_2_/ZrO_2_/Cu:NiOx (homemade)/C (all screen-printed)/(5-AVA)_x_(MA)_1−x_PbI_3_ (drop casting)	12.79/0.8	[101]
											BL-TiO_2_ (spray pyrolysis)/m-TiO_2_/ZrO_2_/C (homemade boron-doped graphite) (all screen-printed)/(5-AVA)_x_(MA)_1−x_PbI_3_ (drop casting)	13.6/No data	[102]
Inkjet printing	jetting voltage, jetting frequency, drop spacing, amount of loaded ink, substrate temperature	ETL, HTL, Perovskite	Low (0.001–0.05)	Up to 40	Up to 50 (1/10th of nozzle diameter)	U: high, W: low	0.1- 1	Up to 10	Up to 6	Limited	BL-TiO_2_ (spray pyrolysis)/m-TiO_2_ (screen printing)/m-ZrO_2_ (screen printing)/MAPI (inkjet printing)/C (screen printing)	9.53/0.16	[134]
											BL-TiO_2_/m-TiO_2_/m-ZrO_2_/MAPI/C All layers were deposited by inkjet printing except carbon layer by screen printing)	9.10/1.5	[135]
Slot-die coating	coating speed, flow rate, coating gap, substrate temperature,	ETL, HTL, Perovskite	Low-High (0.001–5)	Up to 25	Up to 200	U: high, W: low	0.1- 2	Up to 5	No data	Yes	BL-TiO_2_/ZTO/CsFA perovskite/spiro- OMeTAD/C (All layers were deposited by slot die)	9.92/No data	[141]
Blade coating	coating speed, coating gap (distance between blade and substrate), amount of loaded ink, substrate temperature,	ETL, HTL, Perovskite, Carbon	Low (0.001–0.05)	Up to 25	20-few micrometres	U: high, W: moderate	0.1- 10	Up to 2	Up to 1.5 (theoretical data)	Yes	ITO/APTES-linked C_60_ (ETL)/MAPI/C (All layers were deposited by blade coating)	18.64/0.08	[146]
											BL-TiO_2_ (spin coating)/m-TiO_2_ (spin coating)/m-ZrO_2_ (blade coating)/MAPI (drop casting)/NiO NPs (spin coating)/MWCNT as C (electrospray)	15.80/0.08	[151]
Spray coating	nozzle diameter and type, *spray* speed, distance and angle between nozzle and substrate, air or gas pressure, flow rate, substrate temperature	BL, ETL, HTL, Perovskite, Carbon (rarely)	Low-Moderate (0.001–2.5)	Up to 20	20-few µm *(usually 1/10th of nozzle diameter)	U: low to high, W: low to high **	0.05- 1	Up to 12	No data	Yes	BL-TiO_2_/FA_0.85_MA_0.15_PbI_2.85_Br_0.15_/C (All layers were deposited by electrospray)	14.41/0.096	[152]

1-All methods are compatible with commonly used substrates for C-PSCs, i.e., glass and plastic (flexible) substrates. 2-Parameters which should be controlled during printing to obtain desirable thickness and quality of the deposited layer. 3-The most common layers in C-PSCs that can be deposited by the corresponding printing technique. 4-Depending on the layer. * Depending on the spray type. ** Depending on the substrate area and spray system.

## Data Availability

All the data is available within the manuscript.
